# Hospital-at-Home Interventions vs In-Hospital Stay for Patients With Chronic Disease Who Present to the Emergency Department: A Systematic Review and Meta-analysis

**DOI:** 10.1001/jamanetworkopen.2021.11568

**Published:** 2021-06-08

**Authors:** Geneviève Arsenault-Lapierre, Mary Henein, Dina Gaid, Mélanie Le Berre, Genevieve Gore, Isabelle Vedel

**Affiliations:** 1 Lady Davis Institute for Medical Research, Jewish General Hospital, Montréal, Québec, Canada; 2 School of Physical and Occupational Therapy, McGill University, Montréal, Québec, Canada; 3 Université de Montréal, Institut Universitaire de Gériatrie de Montréal, Montréal, Québec, Canada; 4 Schulich Library of Physical Sciences, Life Sciences, and Engineering, McGill University, Montréal, Québec, Canada; 5 Department of Family Medicine, McGill University, Montréal, Québec, Canada

## Abstract

**Importance:**

Hospitalizations are costly and may lead to adverse events; hospital-at-home interventions could be a substitute for in-hospital stays, particularly for patients with chronic diseases who use health services more than other patients. Despite showing promising results, heterogeneity in past systematic reviews remains high.

**Objective:**

To systematically review and assess the association between patient outcomes and hospital-at-home interventions as a substitute for in-hospital stay for community-dwelling patients with a chronic disease who present to the emergency department and are offered at least 1 home visit from a nurse and/or physician.

**Data Sources:**

Databases were searched from date of inception to March 4, 2019. The databases were Ovid MEDLINE, Ovid Embase, Ovid PsycINFO, CINAHL, Health Technology Assessment, the Cochrane Library, OVID Allied and Complementary Medicine Database, the World Health Organization International Clinical Trials Registry Platform, and ClinicalTrials.gov.

**Study Selection:**

Randomized clinical trials in which the experimental group received hospital-at-home interventions and the control group received the usual in-hospital care. Patients were 18 years or older with a chronic disease who presented to the emergency department and received home visits from a nurse or physician.

**Data Extraction and Synthesis:**

Risk of bias was assessed, and a meta-analysis was conducted for outcomes that were reported by at least 2 studies using comparable measures. Risk ratios (RRs) were reported for binary outcomes and mean differences for continuous outcomes. Narrative synthesis was performed for other outcomes.

**Main Outcomes and Measures:**

Outcomes of interest were patient outcomes, which included mortality, long-term care admission, readmission, length of treatment, out-of-pocket costs, depression and anxiety, quality of life, patient satisfaction, caregiver stress, cognitive status, nutrition, morbidity due to hospitalization, functional status, and neurological deficits.

**Results:**

Nine studies were included, providing data on 959 participants (median age, 71.0 years [interquartile range, 70.0-79.9 years]; 613 men [63.9%]; 346 women [36.1%]). Mortality did not differ between the hospital-at-home and the in-hospital care groups (RR, 0.84; 95% CI, 0.61-1.15; *I*^2^ = 0%). Risk of readmission was lower (RR, 0.74; 95% CI, 0.57-0.95; *I*^2^ = 31%) and length of treatment was longer in the hospital-at-home group than in the in-hospital group (mean difference, 5.45 days; 95% CI, 1.91-8.97 days; *I*^2^ = 87%). In addition, the hospital-at-home group had a lower risk of long-term care admission than the in-hospital care group (RR, 0.16; 95% CI, 0.03-0.74; *I*^2^ = 0%). Patients who received hospital-at-home interventions had lower depression and anxiety than those who remained in-hospital, but there was no difference in functional status. Other patient outcomes showed mixed results.

**Conclusions and Relevance:**

The results of this systematic review and meta-analysis suggest that hospital-at-home interventions represent a viable substitute to an in-hospital stay for patients with chronic diseases who present to the emergency department and who have at least 1 visit from a nurse or physician. Although the heterogeneity of the findings remained high for some outcomes, particularly for length of treatment, the heterogeneity of this study was comparable to that of past reviews and further explored.

## Key Points

**Question:**

Are hospital-at-home interventions consisting of, at minimum, home visits from nurses or physicians associated with better patient outcomes for adult patients with a chronic disease who present to an emergency department?

**Findings:**

This systematic review of 9 randomized clinical trial studies, including 959 adult patients with a chronic disease, found that although patients receiving hospital-at-home care had an average length of treatment of 5.4 days longer than that of in-hospital patients and a similar mortality risk, they had a lower risk for readmission by 26% and a lower risk for long-term care admission relative to the in-hospital group. Patients who received hospital-at-home care also had lower depression and anxiety scores than patients receiving in-hospital care, but there was no difference in functional status.

**Meaning:**

This systematic review provides further evidence that hospital-at-home interventions with at least 1 home visit from a nurse or physician may be a promising substitute to in-hospital care, especially for patients with chronic diseases who present to the emergency department.

## Teaser

This meta-analysis investigates the association of hospital-at-home interventions vs in-hospital stay with patient outcomes in adults with chronic disease who present to an emergency department.

## Introduction

Hospitalization is associated with adverse events, nosocomial infections, delirium, and even death^1,2,3,4,5^ and represents important costs for the health care system.^6,7,8^ Furthermore, patients may prefer being cared for at home.^9,10^ Thus, alternatives to hospitalization have been considered.

Hospital-at-home (HaH) interventions were developed to reduce health risks for patients and costs for the system.^11^ These interventions consist of treatment delivered to patients who present with an acute condition; a health care professional provides this treatment in the patient’s home for a condition that would normally require hospitalization.^12,13,14^ In other words, HaH is the delivery of hospital-level care in patients’ homes as a substitute for an in-hospital stay.^15^ Services usually include monitoring, face-to-face clinical care from nurses and physicians, diagnostic testing (eg, laboratory investigations, electrocardiograms, and radiography), and treatment (eg, intravenous medication) in patients’ homes.^15^

Hospital-at-home interventions have attracted widespread interest. A meta-review of HaH interventions has demonstrated its association with better health outcomes and system costs in patients with acute conditions.^16^ However, systematic reviews on complex interventions, like HaH, suffer from high heterogeneity, thereby hindering conclusions made from meta-analyses.^17^

One source of this heterogeneity may be the variability of pooled studies with various interventions and populations.^17^ Systematic reviews often do not distinguish between early discharge^18^ and a substitute for the in-hospital stay altogether.^11,19^ Previous systematic reviews also pooled studies recruiting patients from various entry points (the community, emergency department [ED], and/or during an in-hospital stay).^20^ However, the reasons patients choose to go to the ED rather than visiting their physician vary, one of these being perceived urgency and health care needs.^21^

The interventions’ key components also varied in the systematic reviews, including home visits, phone access, or coordination with home-based services, all of which may influence heterogeneity. Home visits offer an invaluable opportunity to better understand the needs of patients. When carried out by physicians or by nurses collaborating closely with physicians, home visits could provide care that is more consistent with in-hospital care than providing only hospital equipment at home (eg, intravenous therapy) or coordinating home-based services (eg, nurse visits from community services). Furthermore, home visits have been identified as a key component of transitional care and HaH interventions in older patients with chronic diseases.^22^

Hospital-at-home interventions may be particularly fitting for patients with chronic diseases, as these patients tend to use health services more frequently.^23,24,25,26,27,28,29^ Systematic reviews on HaH interventions are usually focused on acute conditions or specific chronic diseases (eg, chronic obstructive pulmonary disease [COPD]) and rarely examine the association of HaH on health outcomes across multiple chronic diseases. Specifically, examining patients with chronic diseases (in consideration of their higher service use than those without chronic diseases) could reduce heterogeneity.

The safety of HaH in terms of patient outcomes, such as mortality and readmission, has been demonstrated.^16^ However, other patient outcomes (eg, patients’ satisfaction, caregiver stress, and out-of-pocket costs) remain inconsistent or unexplored in systematic reviews. In a previous meta-review,^16^ 3 of 6 reviews showed an association between HaH and patient satisfaction, 2 showed no difference, and 1 did not compare patient satisfaction between groups. The reviews that demonstrated an association included studies with various acute conditions, whereas the reviews on specific chronic diseases did not show significant associations.

Given the continuously growing interest in HaH interventions and the high heterogeneity of these complex interventions, it is important to systematically review the literature and assess the association between patient outcomes and HaH interventions considering intervention and population specifics.

The objective of our study was to assess the association between better patient outcomes and HaH interventions aimed at avoiding an in-hospital stay, which included home visits by nurses and/or physicians, for patients with chronic diseases who presented to the ED.

## Methods

### Eligibility Criteria of Included Studies

We conducted a systematic review of the literature guided by the Cochrane Handbook^30^ and the Preferred Reporting Items for Systematic Reviews and Meta-analyses (PRISMA) reporting guideline.^31^ To be included, studies had to be randomized clinical trials (RCTs) that were published in peer-reviewed journals and compared care received in an experimental group (HaH group) with a control group (in-hospital stay group). Hospital-at-home interventions consisted of at least 1 home visit by nurses and/or physicians who provided treatment that would have otherwise been received in the hospital, and in-hospital care consisted of treatment received by patients during an in-hospital stay. To be included, studies had to report at least 1 outcome relating to patients (ie, patient outcomes): clinical (eg, mortality, quality of life, patient or caregiver satisfaction with care, and complications); use of health services (eg, readmission to hospital, out-of-pocket costs); and process (eg, length of treatment). System costs were not considered, because the focus was on patient outcomes. Previous systematic reviews showed that system costs are lower for HaH than for the control group.^16^ Patients included in both groups had to have a chronic disease. Other exclusion criteria are listed in eAppendix 1 in the Supplement. This study did not require institutional review board approval nor was patient consent required, as the systematic review used published, publicly available data.

### Search Strategy, Study Selection, and Data Collection

**Figure 1.**
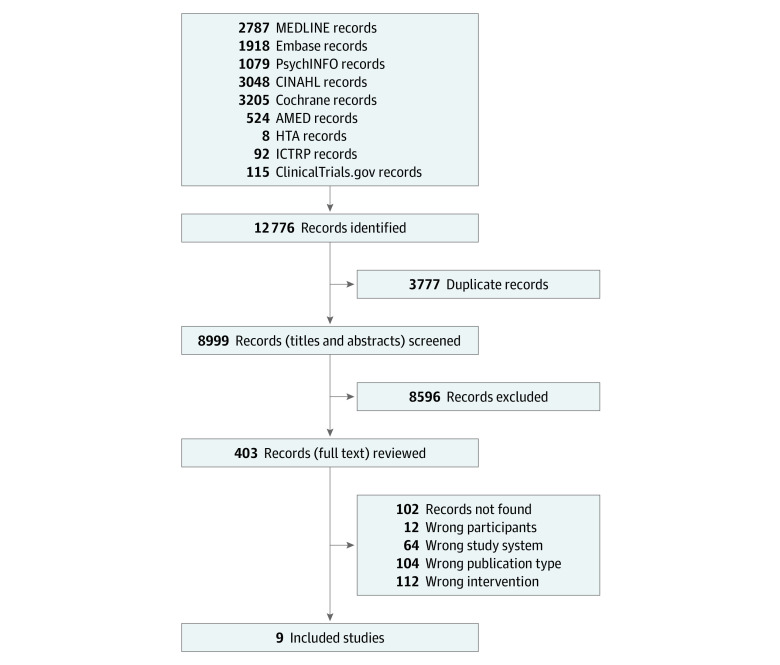
Preferred Reporting Items for Systematic Reviews and Meta-Analyses (PRISMA) Flowchart Search was conducted from the earliest record to March 4, 2019. AMED indicates Allied and Complementary Medicine Database; HTA, Health Technology Assessment; ICTRP, International Clinical Trials Registry Platform.

Three authors (G.A.L., I.V., D.G.) and a health science librarian (G.G.) designed and performed a 3-concept search on March 4, 2019, in 9 databases: Ovid MEDLINE, Ovid Embase, Ovid PsycINFO, CINAHL, Health Technology Assessment, the Cochrane Library, OVID Allied and Complementary Medicine Database, the World Health Organization International Clinical Trials Registry Platform, and ClinicalTrials.gov (Figure 1). The search strategy is outlined in eAppendix 1 in the Supplement.

After removal of duplicates, 2 independent reviewers (D.G., M.H.) screened titles and abstracts, and then they assessed full-text records of potentially eligible studies. Disagreements were resolved by 2 additional reviewers (G.A.L., I.V.). A structured extraction form was developed and piloted on a sample of articles. Data extraction was completed by 1 reviewer (M.H.) and reviewed by a second reviewer (D.G.). Discrepancies were resolved by 2 additional reviewers (G.A.L., I.V.).

Descriptive data were collected for patient characteristics (number of patients, age, proportion of women in each group), characteristics of the interventions, and study design (eg, length of follow-up, home visits by nurses or physicians). The definition of each outcome is provided in eAppendix 1 in the Supplement.

### Risk of Bias

Two reviewers (D.G., M.H.) assessed the risk of bias using criteria from the Cochrane Handbook.^30^ Disagreements were resolved by 2 other reviewers (G.A.L., I.V.). Efforts were made to obtain more information and data (and reduce heterogeneity) by contacting the authors directly, as per Godard-Sebillotte et al.^32^ Details are given in eAppendix 1 in the Supplement.

### Synthesis of Outcomes

Descriptive statistics were conducted on continuous and categorical data, including counts, proportion, CI, mean, median, and SD as appropriate. Meta-analyses were conducted on comparable outcomes measured by at least 2 studies. For binary data, we calculated pooled risk ratio (RR) and 95% CIs. For continuous data, we calculated mean differences and 95% CIs. In both cases, we used a random-effects model to incorporate heterogeneity. Where needed, data transformation was performed (eAppendix 2 in the Supplement). The number of observations used in the meta-analyses was the number of patients at baseline (ie, displayed in flowchart or characteristic table). A 2-sided *P* value less than .05 and a 95% CI that did not cross 1 (RR) or 0 (mean difference) were considered statistically significant. We reported *I*^2^ estimates of heterogeneity. Statistical analyses were performed using the statistical software R, version 1.2.1335 (RStudio Team) and package meta.

We performed sensitivity analyses to assess the robustness of results for each outcome based on suspected modifiers: individual chronic diseases, different follow-up periods, reasons for readmission, sample size, and age of participants. Sensitivity analyses are described in eTables 1 to 4 and eFigures 1 to 3 in the Supplement.

We performed post hoc subgroup analyses to verify whether specific components of the interventions were associated with different results, and we explored reasons for any remaining heterogeneity. We regrouped studies based on home visits by nurses and/or physicians and assessed the magnitude of the association for each outcome.

Outcomes that were not amenable to meta-analysis (eg, reported by 1 study or measured using different tools) were synthesized narratively.^33^ Justifications for performing narrative synthesis are found in eTable 1 in the Supplement.

## Results

### Study Selection

The search identified 8999 records; 8595 were excluded based on title and abstract screening. The remaining 405 records were considered in full text. Of these, 396 records were excluded because the design, publication type, participants, or intervention did not satisfy our criteria or because full text was missing. Reasons for exclusions and the study flowchart are found in Figure 1.

### Risk of Bias

**Figure 2.**
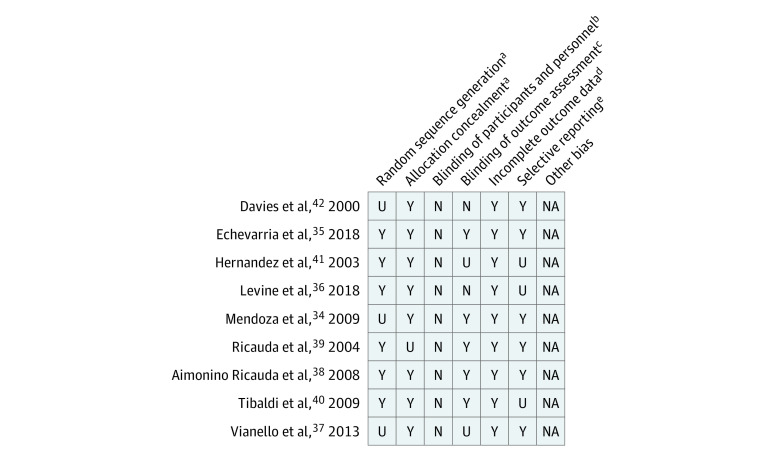
Risk of Bias Quality Appraisal Results Risk of bias was conducted according to the Cochrane Handbook; N indicates no; NA, not applicable; U, unknown; Y, yes. ^a^Selection bias. ^b^Performance bias. ^c^Detection bias. ^d^Attrition bias. ^e^Reporting bias.

We used a 5-criteria of risk of bias appraisal tool (blinding the participants was not possible). Eight studies explicitly concealed allocation from study personnel, 5 studies blinded outcome assessment, 6 studies described random sequence generation, 9 studies presented attrition data, and 6 studies reported complete outcome data. Risk of bias appraisal is presented in Figure 2. Results of efforts to obtain more information and data are described in eAppendix 1 in the Supplement.

### Study Participants and Intervention Characteristics

Nine studies^34,35,36,37,38,39,40,41,42^ were included, providing data on 959 participants (median age, 71.0 years [interquartile range (IQR), 70.0-79.9 years]; 613 men [63.9%] and 346 women [36.1%]) with chronic diseases randomized to either the HaH group or the in-hospital group (Table 1; eAppendix 3 in the Supplement). Median population size was 104 patients (IQR, 71-120 patients) with a median of 52 patients (IQR in HaH group, 37-60 patients vs IQR in in-hospital group, 38-58 patients).^43^ The HaH and in-hospital groups had similar characteristics, except that there were more women in the HaH group than in the in-hospital group (207 of 513 [40.4%] vs 139 of 446 [31.2%], respectively). The study year ranged from 2000 to 2018 and were from 4 different countries (4 studies out of 9 [44.4%] were from Italy).

**Table 1.** Characteristics of Included Studies for HaH and In-Hospital Groups

Source	Design	Patient illness	What and who is involved in the HaH intervention	Outcomes measured	HaH group characteristics^**a**^	In-hospital group characteristics
Mendoza et al,^34^ 2009 Spain	Prospective randomized controlled trial	CHF	Home visits by internal medicine specialist and nurse. Other HCP involved: not specified	Mortality; readmission; functional status; quality of life. Length^b^^,^^c^ of follow-up: 12 mo	37 patients; mean age 78 y; 51% women	34 patients; mean age 80 y; 29% women
Ricauda et al,^38^ 2008 Italy	Prospective randomized controlled single-blind	COPD	Home visits by physicians and nurses. Other HCP involved: geriatricians, physiotherapists, social worker, and counselor	Mortality; morbidity; readmission; depression, functional status, nutritional status, cognitive status; quality of life; caregiver stress; satisfaction. Length of follow-up: 6 mo	52 patients; mean age 80 y; 44% women	52 patients; mean age 79 y; 25% women
Ricauda et al,^39^ 2004 Italy	Randomized, controlled, single-blind trial	Ischemic Stroke	Home visit by nurse, physician, and physical therapist. Other HCP involved: geriatricians, dietitians, physiotherapists, speech therapists, occupational therapists, psychologists, and social workers	Mortality; functional impairment; depression; morbidity; length of treatment; readmission; neurologic deficit. Length of follow-up: 6 mo	60 patients; median age 83 y; 62% women	60 patients; median age 80 y; 48% women
Tibaldi et al,^40^ 2009 Italy	Prospective, single-blind, randomized controlled trial	CHF	Home visits by physician and nurse. Other HCP involved: geriatricians, physiotherapists, social worker, and counselor	Mortality; morbidity readmission; length of treatment; caregiver stress. Length of follow-up: 6 mo	48 patients; mean age 82 y; 54% women	53 patients; mean age 80 y; 43% women
Levine et al,^36^ 2018 United States	Randomized controlled trial	CHF, COPD, or asthma	Home visits by general internist and nurse. Other HCP involved: not specified	Mortality; length of treatment; readmission; morbidity; satisfaction. Length of follow-up: 1 mo	9 patients; median age 65 y; 22% women	11 patients; median age 60 y; 73% women
Davies et al,^42^ 2000 England	Prospective Randomized controlled trial	COPD	Home visits by nurses. Other HCP involved: hospital respiratory physician	Mortality; readmission; quality of life. Length of follow-up: 3 mo	100 patients; mean age 70 y; 55% women	50 patients; mean age 70 y; 40% women
Vianello et al,^37^ 2013 Italy	Prospective Randomized Controlled trial	Neuromuscular disease	Home visit by district nurse, respiratory therapist, or pulmonologist. Other HCP involved: general physician, and trained caregiver	Mortality. Length of follow-up: 3 mo	26 patients; mean age 45 y; 35% women	27 patients; mean age 47 y; 11% women
Hernandez et al,^41^ 2003 Spain	Randomized controlled trial	COPD	Home visit by respiratory nurse. Other HCP involved: respiratory physician	Quality of life; mortality; readmission. Length of follow-up: 2 mo	121 patients; mean age 71 y; 3% women	101 patients; mean age 71 y; 3% women
Echevarria et al,^35^ 2018 England	Noninferiority randomized controlled trial	COPD	Home visits by respiratory specialist nurse. Other HCP involved: respiratory consultant, pharmacist, occupational therapist, physiotherapist, and social support	Mortality; readmission; depression and anxiety; quality of life; length of treatment. Length of follow-up: 3 mo	60 patients; mean age 71 y; 53% women	58 patients; mean age 69 y; 52% women

Abbreviations: CHF, chronic heart failure; COPD, chronic obstructive pulmonary disease; HaH, hospital-at-home; HCP, health care professional.

^a^ Number of patients counted at baseline.

^b^ Length of treatment defined as number of days in HaH for the experimental group and the number of in-hospital days for the control group.

^c^ Length of follow-up was defined as the number of months for which outcome data was collected for both HaH and in-hospital groups.

All studies included home visits by nurses, and 5 studies^34,36,38,39,40^ included home visits by nurses and/or physicians (all were hospital or HaH team staff). Additional intervention components included phone access and availability (7 studies^35,36,37,38,39,40^), patient and caregiver education (3 studies^38,40,41^), social services (4 studies^39,40,41,42^), and household support (2 studies^35,41^). Some studies included additional staff on their HaH team, such as social workers (3 studies^38,39,40^), respiratory therapists (2 studies^35,37^), occupational therapists (2 studies^35,39^), physiotherapists (4 studies^35,38,39,40^), dieticians (1 study^39^), speech therapists (1 study^39^), and pharmacists (1 study^35^). The median follow-up period was 3 months (IQR, 2-6 months) varying from 1 to 12 months.

### Results of Meta-analyses

Outcomes analyzed via meta-analysis were mortality (all 9 studies^34,35,36,37,38,39,40,41,42^), readmission (7 studies^34,35,36,38,40,41,42^), length of treatment (5 studies^34,35,38,39,40^), and long-term care admission (3 studies^38,39,40^). For all outcomes, we used the longest follow-up period, because intermediate time points were not amenable to meta-analysis. Although 2 studies^35,42^ provided more than 1 time point data for mortality (14 and 90 days), 1 study^35^ counted 0 mortality at 14 days for both groups, making it not amenable to meta-analysis. Forest plots are presented in eAppendix 4 in the Supplement.

There was no significant difference between the HaH and in-hospital groups in mortality (RR, 0.84; 95% CI, 0.61-1.15). There was a lower risk for readmission in the HaH group than in the in-hospital group (RR, 0.74; 95% CI, 0.57-0.95). Length of treatment was significantly longer in the HaH group than in the in-hospital group (mean difference, 5.4 days; 95% CI, 1.9-9.0 days). There was a statistically significantly lower risk of long-term care admission in the HaH group than in the in-hospital group (RR, 0.16; 95% CI, 0.03-0.74) (Table 2; eAppendix 4 in the Supplement). Heterogeneity (*I*^2^) was 0% for mortality and long-term care admission, 31% for readmission, and 87% for length of treatment (eAppendix 4 in the Supplement).

**Table 2.** Meta-analysis Comparing HaH and In-Hospital Groups

Outcome	HaH group	In-hospital group	Risk ratio or mean difference (95% CI)	95% Prediction interval	*P* value
Mortality					
No. of observations	513	446	0.84 (0.61 to 1.15)	0.57 to 1.24	.28
No. of events (%)	57 (11.1)	63 (14.1)			
Readmission					
No. of observations	427	359	0.74 (0.57 to 0.95)^a^	0.41 to 1.32	.02
No. of events (%)	123 (28.8)	139 (38.7)			
Length of treatment					
No. of observations	257	257	5.45 (1.91 to 8.98)^a^	−7.30 to 18.19	.003
Mean (SD), d	18 (12.6)	11 (6.9)			
Long-term care admission					
No. of observations	160	165	0.16 (0.03 to 0.74)^a^	NA	.02
No. of events (%)	1 (0.6)	16 (9.7)			

Abbreviations: HaH, hospital-at-home; NA, not applicable.

^a^ Significant result.

We analyzed individual chronic diseases in sensitivity analyses and did not find a significant difference between the HaH and in-hospital groups in readmission for patients with only COPD or chronic heart failure (CHF). However, the direction of the associations and magnitude remained comparable. Similarly, we did not find a significant difference between the HaH and in-hospital groups in length of treatment for patients with only CHF, although the direction and magnitude of the associations remained comparable. When considering various lengths of follow-up periods in sensitivity analyses, we did not find a significant difference between the HaH and in-hospital groups on readmission at 3-month follow-up. All other sensitivity analyses (age, population size, and reasons for readmission) yielded similar results as the original analyses.

We performed post hoc subgroup analyses on specific components of the intervention (home visits by nurses and/or physicians). The 4 studies^35,37,41,42^ in which home visits were performed by nurses alone did not seem to differ from the 5 studies^34,36,38,39,40^ in which home visits were performed by nurses and physicians. The magnitude of the RR estimates for mortality in studies with physician visits ranged from 0.6 to 1.12, whereas that of nurses-only studies ranged from 0.6 to 0.97. Two nurses-only studies reported on readmission, with RR estimates of 0.74 and 1.09, whereas that of studies with physicians visits ranged from 0.31 to 0.81. This analysis was not conducted on length of treatment because only 1 nurse-only study reported on this outcome, with similar results in both groups.

### Narrative Synthesis

Outcomes synthesized narratively included anxiety and depression, quality of life, patient satisfaction, caregiver stress, cognitive status, nutrition, morbidity due to hospitalization, functional status, and neurological deficits. Most outcomes were measured at longest follow-up period, except for 1 study,^35^ which reported intermediate time points at 14 days for anxiety and quality-of-life outcomes. Results are presented in Table 3 and eAppendix 3 in the Supplement.

**Table 3.** Summary of Outcomes Synthesized Narratively

Variable	Measurement tools or outcomes	Study conclusions
Cognitive status	Mini Mental State Exam^38^	No difference
Nutrition	Mini Nutritional Assessment Tool^38^	No difference
Patient satisfaction	Unidentified questionnaire^38^	No difference
	“Patient experience” as measured by a composite score including 2 tools: Care Transition Measure 3 and Picker Patient Experience and 2 questions: whether participant recommend the hospital and how they rate their global experience^36^	No difference
	Unidentified questionnaire^41^	Slightly higher in HaH patients compared to in-hospital patients
	Single question to assess whether the patient would prefer HaH^35^	Both HaH and in-hospital patients preferred or would have preferred HaH
Morbidity due to hospitalization	Morbidity (ie, urinary tract infections, catheterization, falls, delirium, pressure sores)^38^	Less urinary tract infections in the HaH group compared to the in-hospital group; no other differences
	Respiratory infections and urinary tract infections^39^	No difference
	Adverse events^36^	One adverse event in the in-hospital patients compared with none in the HaH patients
	Morbidity (infections, delirium, bed sores, deep vein thrombosis, and falls)^40^	Slightly lower in HaH patients compared with in-hospital patients (not statistically significant)
Caregiver stress	Relative Stress Scale^38,40^	One study^38^ found no difference in the change between in-hospital and HaH patients. The other study^40^ found caregiver stress of HaH patients decreased at discharge, but was higher at admission
Anxiety and depression	Hospital Anxiety and Depression Scale^35^	HaH patients showed improvement for anxiety at 14 d, not at 90 d, follow-up whereas in-hospital patients worsened
	Geriatric Depression Scale^38,39^	More improvement in HaH patients compared with in-hospital patients
Quality of life	Short Form Health Surveys-36^34^and 12^41^	No difference
	Nottingham Health Profile^38^	More improvement in HaH patients compared with in-hospital patients
	St George’s Respiratory Questionnaire^41,42^	One study^42^ found no difference. The other study^41^ found that HaH patients improved more than in-hospital patients
	EuroQuality of Life Instrument 5D-5L^35^	More improvement in HaH and in-hospital patients at 14 d; no difference at 90 d
Functional status	Barthel Index^34^	No difference
	Katz Instrument for Activities of Daily Living and Lawton Instrumental Activities of Daily Living^38^	No difference in either instruments
	7-item Functional Impairment Measure and Activities of Daily Living^39^	No difference in either instruments
Neurologic deficit	Canadian Neurological scale^39^	No difference
	National Institutes Health Stroke Scale score^39^	No difference

Abbreviation: HaH, hospital-at-home.

All 3 studies^35,38,39^ looking at anxiety and depression reported that it improved more in the HaH group than the in-hospital group. Five studies that evaluated quality of life reported mixed findings: 3 studies^35,38,41^ found that it improved more in the HaH group than in the in-hospital group, and 2 studies^34,41^ found no difference. Three studies that evaluated patient satisfaction reported mixed results: 1 study^41^ found a higher patient satisfaction in the HaH group than in the in-hospital group, whereas 2 studies^36,38^ showed no difference. Two studies that evaluated caregiver stress reported mixed results: one^40^ found higher stress at admission that decreased at discharge in the HaH group, whereas caregiver stress did not change in the in-hospital group. The other study^38^ found no difference. All 3 studies that evaluated functional status found no difference between the groups.^34,38,39^ No study reported out-of-pocket costs for patients or caregivers, and 4 studies^36,38,39,40^ that evaluated morbidity due to hospitalization reported mixed results.

## Discussion

In this systematic review and meta-analysis, study results suggest that patients with chronic diseases who presented to the ED and were treated with HaH interventions had a lower risk of hospital readmission and long-term care admission than those who received in-hospital care. We found no difference in mortality between the 2 groups, but we found that length of treatment was longer in the HaH group than in the in-hospital group. Taken together, our findings suggest that for patients with chronic diseases who present to the ED, HaH interventions may be as safe as hospitalization (with no difference in mortality) and a preferred alternative (with lower risk of readmission). Furthermore, we found that HaH intervention may be associated with better anxiety and depression scores but not with functional status.

The results of our meta-analysis are consistent with those of other systematic reviews that found lower risk of readmission^19,44^ and no difference in risk of mortality.^15,45^ Since the writing of our manuscript, a new RCT was published and reported similar results.^46^

The results from our narrative synthesis for lower anxiety and depression were also similar to previous systematic reviews.^15,20^ Although another review article that evaluated various medical conditions has shown better patient satisfaction for HaH interventions than that of their control,^16^ we found mixed results. This was probably due to the variety of assessment tools measuring different concepts of satisfaction.

Although costs related to the health care system have been shown to be lower for HaH interventions than for in-hospital care,^16^ none of the studies in our review reported out-of-pocket costs. It is possible that in HaH interventions, some costs are transferred to patients and caregivers.^47,48^ Considering the longer length of treatment in the HaH group, it will be important to assess out-of-pocket costs in future studies.

### Recommendations for Future Studies

Our results suggest various ways that future RCTs on HaH interventions may improve. First, more RCTs should evaluate the association between patient outcomes and HaH intervention in patients with chronic disease who present to the ED by using standard outcomes and measurements. It will be important to report out-of-pocket costs to gain a better understanding of what HaH interventions actually cost, especially given the longer length of treatment experienced in the HaH patient group. Randomized clinical trials should clearly define their interventions and report on process outcomes to allow further exploration of factors that may contribute to different results. Finally, studies should also consider sex-based bias in these HaH studies.

### Limitations

This study has some limitations, particularly regarding potential sources of heterogeneity. Despite efforts to reduce heterogeneity by selecting studies with specific intervention components (hospital avoidance, recruitment from the ED, home visits by nurses or physicians) and specific patient characteristics (chronic diseases), we still observed high heterogeneity, especially for length of treatment. The heterogeneity in our meta-analyses was similar to what was found in other reviews, where it varied between 0% and 1%^15,44,49,50^ for mortality, between 17% and 45%^15,44,49,50^ for readmission, and 88% for length of treatment.^15^ The heterogeneity of our findings may be explained by other characteristics related to the intervention, population, and outcomes, as well as the context in which the interventions were implemented and the studies conducted.^17^

Despite selecting specific components of the interventions, variations remained across studies in terms of home visits by hospital or HaH team nurses alone or by nurses and physicians. However, the magnitude of the association in studies with or without physician home visits did not appear to differ, especially for mortality and readmission. Other components of the interventions varied across studies (eg, phone calls, other health professional consultations, home support, education) and may contribute to heterogeneity. Further studies should explore other components of interventions.

Variations in the patients’ characteristics may have contributed to the heterogeneity of our findings. Although most of the patients included had either COPD or CHF (4 studies included only patients with COPD, and 2 studies included only patients with CHF), sensitivity analyses limited to either COPD or CHF no longer yielded a significant association in terms of readmission and length of treatment. Although the significance is different in the sensitivity analyses compared with that of the original analyses, the direction and magnitude of the associations remained comparable. This difference in significance may have been due to the small number of studies in the sensitivity analyses. Pooling studies conducted with patients with different chronic diseases may not be sufficient to reduce variability in the patients' characteristics, especially considering the various clinical criteria for admission owing to the specifics of the patients’ diseases. Only 1 RCT evaluated patients with different chronic diseases. However, there is an intrinsic interest in monitoring this population of patients, because they are high users of health services compared with patients without chronic diseases.^26,28,51^

Most patients in our review were older; removing the 1 study with younger patients did not alter our results. Women were underrepresented in our study compared with the proportion of older women globally. Furthermore, the proportion of women varied between studies as well as within studies. This may have been an important source of heterogeneity, because men use hospital services more than women.^52^ Further research regarding these findings is needed.

The operationalization of outcomes poses challenges to all systematic reviews; ours was no exception. This was especially true for the length of treatment. It was the only process outcome in our study, but it was neither clearly defined nor referred to consistently (eg, length of stay, length of treatment, time to recovery). Systematic reviews often do not report the pooled results for length of treatment for these reasons. We pooled the length of treatment in our paper nonetheless, as we think that this high heterogeneity is not a sufficient rationale, especially in the context of complex interventions such as HaH.^17^

Considering process outcomes is important in the evaluation of interventions because it allows for the exploration and explanation of underlying factors associated with the success or lack thereof of an intervention.^53^ Process outcomes may provide valuable information on the heterogeneity between and within studies. We found that the HaH group experienced a longer length of treatment than the in-hospital group. This is important to note, as one likely benefit of HaH is the smoother transition between hospital and home. In fact, many components of HaH are similar to transitional care interventions, such as multidisciplinary approaches and close monitoring, which have been shown to reduce readmission in patients with chronic diseases.^22,54^ Our efforts to obtain clarification for this outcome were answered by 1 study.^35^

Other possible variations in outcomes consisted of differing follow-up periods. Our sensitivity analyses suggest that among studies with a 3-month follow-up, there was no longer a significant difference in hospital readmission between the HaH and in-hospital groups. This sensitivity analysis was limited to only 2 studies^35,42^ and will require future studies.

Another source of heterogeneity concerns the context in which the HaH interventions were implemented and the context in which the studies were conducted. We found a wide range in publication year (2000 to 2018) and country of origin (many from Italy). In future studies, this variability in contexts should be analyzed further, as hospitals and available technologies have evolved considerably over time and are unique to specific contexts.

Overall, the small number of studies in our review limits a deeper examination of heterogeneity. However, we conducted random-effects models to incorporate this heterogeneity. Neither selecting studies with specific intervention components nor looking at specific patient characteristics seemed to change our findings’ statistical heterogeneity. However, we generated hypotheses for heterogeneity based on variations in interventions, population characteristics, outcome definitions, and study context.

## Conclusions

The results of our systematic review support the use of HaH interventions in people with chronic disease. Given the current global COVID-19 pandemic wherein risk of infectious disease spread is a major concern, especially for patients with chronic diseases, HaH may be considered as a viable alternative to hospitalization.^55^

## Notes

Supplement. **eAppendix 1.** Methods Details **eAppendix 2.** Data Transformation **eTable 1.** Justification for Narrative Synthesis **eTable 2.** Justification for Sensitivity Analyses for Mortality **eFigure 1.** Forest Plots for Mortality Sensitivity Analyses **eTable 3.** Justification of Sensitivity Analyses for Readmission **eFigure 2.** Forest Plots for Readmission Sensitivity Analyses **eTable 4.** Justification of Sensitivity Analyses for Length of Treatment **eFigure 3.** Forest Plots for Length of Treatment Sensitivity Analyses **eAppendix 3.** Summary of Findings Table **eAppendix 4.** Forest Plots of Original Meta-Analyses eReferences
