# Supplementary material for: Hospital-at-Home Interventions vs In-Hospital Stay for Patients With Chronic Disease Who Present to the Emergency Department: A Systematic Review and Meta-analysis
Source: JAMA Netw Open. 2021 Jun 8;4(6):e2111568. doi: 10.1001/jamanetworkopen.2021.11568 (PMC8188269; doi:10.1001/jamanetworkopen.2021.11568)
Supplement: Supplement. — eAppendix 1. Methods Details eAppendix 2. Data Transformation eTable 1. Justification for Narrative Synthesis eTable 2. Justification for Sensitivity Analyses for Mortality eFigure 1. Forest Plots for Mortality Sensitivity Analyses eTable 3. Justification of Sensitivity Analyses for Readmission eFigure 2. Forest Plots for Readmission Sensitivity Analyses eTable 4. Justification of Sensitivity Analyses for Length of Treatment eFigure 3. Forest Plots for Length of Treatment Sensitivity Analyses eAppendix 3. Summary of Findings Table eAppendix 4. Forest Plots of Original Meta-Analyses eReferences [file jamanetwopen-e2111568-s001.pdf]

Supplementary Online Content

Arsenault-Lapierre G, Henein M, Gaid D, Le Berre M, Gore G, Vedel I. Hospital-at-home interventions vs in-hospital stay for patients with chronic disease who present to the emergency department: a systematic review and meta-analysis. *JAMA Netw Open*. 2021;4(6):e2111568. doi:10.1001/jamanetworkopen.2021.11568

eAppendix 1. Methods Details

eAppendix 2. Data Transformation

eTable 1. Justification for Narrative Synthesis

eTable 2. Justification for Sensitivity Analyses for Mortality

eFigure 1. Forest Plots for Mortality Sensitivity Analyses

eTable 3. Justification of Sensitivity Analyses for Readmission

eFigure 2. Forest Plots for Readmission Sensitivity Analyses

eTable 4. Justification of Sensitivity Analyses for Length of Treatment

eFigure 3. Forest Plots for Length of Treatment Sensitivity Analyses

eAppendix 3. Summary of Findings Table

eAppendix 4. Forest Plots of Original Meta-Analyses

eReferences

This supplementary material has been provided by the authors to give readers additional information about their work.

© 2021 Arsenault-Lapierre G et al. *JAMA Network Open.*

eAppendix 1. Methods Details

Exclusion criteria

Studies with obstetric, mental health, and rehabilitation populations were excluded. Long-term care, post-surgery, services provided in outpatient settings (not within patients' homes), post-discharge interventions, end-of-life care at home, and self-care by the patient in their home, such as self-administration of an intravenous infusion, were excluded.

Detailed electronic search strategy

We performed a 3-concept search using combinations of subject headings and text words for the concepts of "Hospital-at-Home", "ED", and "randomized controlled trials", from inception to March 4, 2019, with no language restriction.

Original Searches
****

1. **Database: Ovid MEDLINE(R) and Epub Ahead of Print, In-Process & Other Non-Indexed Citations, Ovid MEDLINE(R) Daily <1946 to Present>**

Search strategy:
----

1 (hospital* adj2 at home).tw,kf. (1132)
2 Home Care Services, Hospital-Based/ (1834)
3 (home* versus hospital* or home care versus hospital* or home* vs hospital* or home care vs hospital* or hospital* versus home* or hospital* vs home* or "home* or hospital*" or "home care or hospital*" or "hospital* or home*").tw,kf. (612)
4 Home hospitali#ation*.tw,kf. (182)
5 ((hospital* or conventional care or conventional management or conventional therap* or conventional treatment* or usual care) adj2 alternative*).tw,kf. (1255)
6 (admission* adj2 avoid*).tw,kf. (616)
7 ((Home-based or "at home" or home care or homecare or home treatment* or home therap* or ((early or earlier or home*) adj2 discharge*) or ((outpatient or out patient) adj (setting* or care))) adj15 (hospital-based or hospital care or in hospital or ((inpatient or in patient) adj (care or setting*)) or general ward$1 or hospitaliz* or hospitalis* or usual care or conventional care or conventional management or conventional hospital* or conventional therap* or conventional treatment*)).tw,kf. (6713)
8 or/2-7 (10499)
9 exp emergency health services, hospital/ or ambulatory care/ or exp community health services/ (324355)
10 (emergenc* or acute or urgent care or community or outpatient* or out patient* or ambulatory or clinic or clinics or er or ed or ers or eds or "at hospital" or home care or homecare).mp. (2594752)
11 9 or 10 (2726732)

© 2021 Arsenault-Lapierre G et al. *JAMA Network Open.*

12 8 and 11 (8066)
13 1 or 12 (8689)
14 ((randomized controlled trial or controlled clinical trial).pt. or randomized.ab. or randomised.ab. or placebo.ab. or drug therapy.fs. or randomly.ab. or trial.ab. or groups.ab.) not (exp animals/ not humans.sh.) (3805620)
14 13 and 14 (2787)

****

2. Database: Embase Classic+Embase <1947 to 2019 March 01>

Search strategy:

----

1 (hospital* adj2 at home).tw,kf. (1751)
2 (home* versus hospital* or home care versus hospital* or home* vs hospital* or home care vs hospital* or hospital* versus home* or hospital* vs home* or "home* or hospital*" or "home care or hospital*" or "hospital* or home*").tw,kf. (870)
3 Home hospitali#ation*.tw,kf. (263)
4 ((hospital* or conventional care or conventional management or conventional therap* or conventional treatment* or usual care) adj2 alternative*).tw,kf. (1691)
5 (admission* adj2 avoid*).tw,kf. (1121)
6 ((Home-based or "at home" or home care or homecare or home treatment* or home therap* or ((early or earlier or home*) adj2 discharge*) or ((outpatient or out patient) adj (setting* or care))) adj15 (hospital-based or hospital care or in hospital or ((inpatient or in patient) adj (care or setting*)) or general ward$1 or hospitaliz* or hospitalis* or usual care or conventional care or conventional management or conventional hospital* or conventional therap* or conventional treatment*)).tw,kf. (10763)
7 or/2-6 (14199)
8 emergency ward/ or exp emergency treatment/ or exp ambulatory care/ or exp community care/ (521155)
9 (emergenc* or acute or urgent care or community or outpatient* or out patient* or ambulatory or clinic or clinics or er or ed or ers or eds or "at hospital" or home care or homecare).mp. (3760226)
10 8 or 9 (3924702)
11 7 and 10 (10218)
12 1 or 11 (11311)
13 crossover-procedure/ or double-blind procedure/ or randomized controlled trial/ or single-blind procedure/ or (random* or factorial* or crossover* or cross over* or placebo* or (doubl* adj blind*) or (singl* adj blind*) or assign* or allocat* or volunteer*).tw. (2140996)
14 12 and 13 (1918)

****

3. Database: PsycINFO <1806 to February Week 4 2019>

Search Strategy:

----

1 (hospital* adj2 at home).mp. (172)

© 2021 Arsenault-Lapierre G et al. *JAMA Network Open.*

2 (home* versus hospital* or home care versus hospital* or home* vs hospital* or home care vs hospital* or hospital* versus home* or hospital* vs home* or "home* or hospital*" or "home care or hospital*" or "hospital* or home*").mp. (164)
3 Home hospitali#ation*.mp. (19)
4 ((hospital* or conventional care or conventional management or conventional therap* or conventional treatment* or usual care) adj2 alternative*).mp. (368)
5 (admission* adj2 avoid*).mp. (76)
6 ((Home-based or "at home" or home care or homecare or home treatment* or home therap* or ((early or earlier or home*) adj2 discharge*) or ((outpatient or out patient) adj (setting* or care))) adj15 (hospital-based or hospital care or in hospital or ((inpatient or in patient) adj (care or setting*)) or general ward$1 or hospitaliz* or hospitalis* or usual care or conventional care or conventional management or conventional hospital* or conventional therap* or conventional treatment*)).mp. (1553)
7 or/2-6 (2076)
8 exp emergency services/ or exp outpatient treatment/ or exp community services/ (44649)
9 (emergenc* or acute or urgent care or community or outpatient* or out patient* or ambulatory or clinic or clinics or er or ed or ers or eds or "at hospital" or home care or homecare).mp. (512502)
10 8 or 9 (516675)
11 7 and 10 (1554)
12 1 or 11 (1666)
13 (control* or random* or placebo* or double blind*).mp. or exp treatment/ (1365282)
14 12 and 13 (1079)

****

4. CINAHL

Search strategy:

#	Query	Limiters/Expanders	Last Run Via	Results
S19	S17 AND S18	Search modes - Boolean/Phrase	Interface – EBSCOhost Research Databases Search Screen - Advanced Search Database - CINAHL Plus with Full Text	3,048
S18	(MH "randomized controlled trials" OR MH "double-blind studies" OR MH "single-blind studies"	Search modes - Boolean/Phrase	Interface – EBSCOhost Research Databases Search Screen - Advanced Search Database - CINAHL Plus with	542,221

© 2021 Arsenault-Lapierre G et al. *JAMA Network Open.*

	OR MH "random assignment" OR MH "pretest-posttest design" OR MH "cluster sample" OR TI (randomised OR randomized) OR AB (random*) OR TI (trial) OR (MH "sample size" AND AB (assigned OR allocated OR control)) OR MH "placebos" OR PT "randomized controlled trial" OR AB (control W5 group) OR MH "crossover design" OR MH "comparative studies" OR AB (cluster W3 RCT)) NOT ((MH "animals+" OR MH "animal studies" OR TI (animal model*)) NOT MH "human")			
S17	S1 OR S16	Search modes - Boolean/Phrase	Interface - EBSCOhost Research Databases Search Screen - Advanced Search Database - CINAHL Plus with Full Text	14,985
S16	S7 AND S15	Search modes - Boolean/Phrase	Interface - EBSCOhost Research Databases Search Screen - Advanced	14,614

© 2021 Arsenault-Lapierre G et al. *JAMA Network Open.*

			Search Database - CINAHL Plus with Full Text	
S15	S8 OR S9 OR S10 OR S11 OR S12 OR S13 OR S14	Search modes - Boolean/Phrase	Interface - EBSCOhost Research Databases Search Screen - Advanced Search Database - CINAHL Plus with Full Text	1,242,679
S14	(MH "Outpatients")	Search modes - Boolean/Phrase	Interface - EBSCOhost Research Databases Search Screen - Advanced Search Database - CINAHL Plus with Full Text	42,637
S13	(MH "Outpatient Service")	Search modes - Boolean/Phrase	Interface - EBSCOhost Research Databases Search Screen - Advanced Search Database - CINAHL Plus with Full Text	7,471
S12	(emergenc* or acute or "urgent care" or community or outpatient* or "out patient*" or ambulatory or clinic or clinics or er or ed or ers or eds or "at hospital" or "home care" or homecare)	Search modes - Boolean/Phrase	Interface - EBSCOhost Research Databases Search Screen - Advanced Search Database - CINAHL Plus with Full Text	991,121
S11	(MH "Emergency Medical Services+")	Search modes - Boolean/Phrase	Interface - EBSCOhost Research Databases Search Screen - Advanced Search Database - CINAHL Plus with Full Text	88,008
S10	(MH "Community Health Services+")	Search modes - Boolean/Phrase	Interface - EBSCOhost	377,143

© 2021 Arsenault-Lapierre G et al. *JAMA Network Open.*

			Research Databases Search Screen - Advanced Search Database - CINAHL Plus with Full Text	
S9	(MH "Ambulatory Care")	Search modes - Boolean/Phrase	Interface - EBSCOhost Research Databases Search Screen - Advanced Search Database - CINAHL Plus with Full Text	10,610
S8	(MH "Emergency Service")	Search modes - Boolean/Phrase	Interface - EBSCOhost Research Databases Search Screen - Advanced Search Database - CINAHL Plus with Full Text	45,423
S7	S2 OR S3 OR S4 OR S5 OR S6	Search modes - Boolean/Phrase	Interface - EBSCOhost Research Databases Search Screen - Advanced Search Database - CINAHL Plus with Full Text	15,628
S6	(("Home-based" or "at home" or "home care" or homecare or "home treatment*" or "home therap*" or ((early or earlier or home*) N2 discharge*) or ((outpatient or "out patient") N1 (setting* or care))) N15 ("hospital-based" or "hospital care" or "in hospital" or ((inpatient or "in patient") N1 (care or setting*)) or "general ward*" or hospitaliz* or hospitalis* or "usual care" or "conventional care" or "conventional	Search modes - Boolean/Phrase	Interface - EBSCOhost Research Databases Search Screen - Advanced Search Database - CINAHL Plus with Full Text	14,347

© 2021 Arsenault-Lapierre G et al. *JAMA Network Open.*

	management" or "conventional hospital*" or "conventional therap*" or "conventional treatment*"))			
S5	(admission* N2 avoid*)	Search modes - Boolean/Phrase	Interface - EBSCOhost Research Databases Search Screen - Advanced Search<br>Database - CINAHL Plus with Full Text	443
S4	((hospital* or "conventional care" or "conventional management" or "conventional therap*" or "conventional treatment*" or "usual care") N2 alternative*)	Search modes - Boolean/Phrase	Interface - EBSCOhost Research Databases Search Screen - Advanced Search<br>Database - CINAHL Plus with Full Text	633
S3	(home hospitalisation* OR home hospitalization*)	Search modes - Boolean/Phrase	Interface - EBSCOhost Research Databases Search Screen - Advanced Search<br>Database - CINAHL Plus with Full Text	54
S2	(home* versus hospital* or home care versus hospital* or home* vs hospital* or home care vs hospital* or hospital* versus home* or hospital* vs home* or "home* or hospital*" or "home care or hospital*" or "hospital* or home*")	Search modes - Boolean/Phrase	Interface - EBSCOhost Research Databases Search Screen - Advanced Search<br>Database - CINAHL Plus with Full Text	3,011
S1	(hospital* N2 "at home")	Search modes - Boolean/Phrase	Interface - EBSCOhost Research Databases Search Screen -	5,115

© 2021 Arsenault-Lapierre G et al. *JAMA Network Open.*

			Advanced Search<br>Database - CINAHL Plus with Full Text	

****

5. **Cochrane Library – Reviews, Protocols, Trials**

Date Run: 04/03/2019 17:46:04

Search strategy:

ID Search Hits

#1 (hospital* NEAR/2 "at home"):ti,ab,kw 1267

#2 (home* next versus next hospital* or home next care next versus next hospital* or home* next vs next hospital* or home next care next vs next hospital* or hospital* next versus next home* or hospital* next vs next home* or "home or" next hospital* or "home care or" next hospital* or hospital* next "or home"):ti,ab,kw 216

#3 (home next hospitalisation* OR home next hospitalization*):ti,ab,kw 32

#4 ((hospital* or conventional next care or conventional next management or conventional next therap* or conventional next treatment* or usual next care) NEAR/2 alternative*):ti,ab,kw 153

#5 (admission* NEAR/2 avoid*):ti,ab,kw 55

#6 ((Home next based or at next home or home next care or homecare or home next treatment* or home next therap* or ((early or earlier or home*) NEAR/2 discharge*) or ((outpatient or out next patient) NEAR/1 (setting* or care))) NEAR/15 (hospital next based or hospital next care or in next hospital or ((inpatient or in next patient) NEAR/1 (care or setting*)) or general next ward* or hospitaliz* or hospitalis* or usual next care or conventional next care or conventional next management or conventional next hospital* or conventional next therap* or conventional next treatment*)):ti,ab,kw 2812

#7 #2 OR #3 OR #4 OR #5 OR #6 3046

#8 (emergenc* or acute or urgent next care or community or outpatient* or out next patient* or ambulatory or clinic or clinics or er or ed or ers or eds or "at hospital" or home next care or homecare):ti,ab,kw 271889

#9 #7 AND #8 2646

#10 #1 OR #9 in Cochrane Reviews, Cochrane Protocols, Trials 3205

****

6. **Database: AMED (Allied and Complementary Medicine) <1985 to March 2019>**

Search Strategy:

----

1 (hospital* adj2 at home).mp. (441)

2 (home* versus hospital* or home care versus hospital* or home* vs hospital* or home care vs hospital* or hospital* versus home* or hospital* vs home* or "home* or hospital*" or "home care or hospital*" or "hospital* or home*").mp. (317)

3 Home hospitali#ation*.mp. (9)

4 ((hospital* or conventional care or conventional management or conventional therap* or conventional treatment* or usual care) adj2 alternative*).mp. (70)

© 2021 Arsenault-Lapierre G et al. *JAMA Network Open.*

5 (admission* adj2 avoid*).mp. (12)
6 ((Home-based or "at home" or home care or homecare or home treatment* or home therap* or ((early or earlier or home*) adj2 discharge*) or ((outpatient or out patient) adj (setting* or care))) adj15 (hospital-based or hospital care or in hospital or ((inpatient or in patient) adj (care or setting*)) or general ward$1 or hospitaliz* or hospitalis* or usual care or conventional care or conventional management or conventional hospital* or conventional therap* or conventional treatment*)).mp. (2193)
7 or/2-6 (2314)
8 (emergenc* or acute or urgent care or community or outpatient* or out patient* or ambulatory or clinic or clinics or er or ed or ers or eds or "at hospital" or home care or homecare).mp. (91081)
9 7 and 8 (2286)
10 1 or 9 (2301)
11 (control* or random* or placebo* or double blind*).mp. or trial.ti. (45157)
12 10 and 11 (524)

****

7. HTA

----

http://www.crd.york.ac.uk/PanHTA/ResultsPage.asp
Canadian and International HTA
March 4, 2019
Search strategy:
"hospital at home" OR "hospitalization at home" OR "hospitalisation at home" OR "home hospitalization" OR "home hospitalizations" OR "home hospitalisation" OR "home hospitalisations"
8 results

****

8. International Clinical Trials Registry Platform (ICTRP) – WHO

----

http://apps.who.int/trialsearch/default.aspx
March 4, 2019
Search strategy:
hospital at home OR home hospitalization OR home hospitalisation OR home hospital
92 results (98 trials)

****

9. ClinicalTrials.gov

----

https://clinicaltrials.gov/
March 4, 2019
Search strategy:
"hospital at home" OR "home hospitalization" OR "home hospitalisation" OR "home hospital" OR (home AND hospital)
115 results

© 2021 Arsenault-Lapierre G et al. *JAMA Network Open.*

****
****

Outcome definitions

Mortality: Mortality was defined as the total number of deaths recorded at the available time points for each study.

Readmission: Readmission was defined as the number of patients who were admitted (HaH) or readmitted (in-hospital group) to the hospital at the available time points of each study. All-cause readmissions were considered, except for one study that reported readmissions due only to the disease of the population (Chronic Heart Failure, CHF)(Mendoza et al. 2009).

Length of treatment: Length of treatment was defined as the number of days in HaH care for the experimental group and the number of in-hospital days at the index admission for the control group.

Long-term care admission: Admission to long-term care was defined as the number of patients admitted to long-term care or institutionalized at the available time points.

Anxiety and depression: Anxiety and depression was defined as the change in depression and/or anxiety scores from baseline to the available time points.

Quality-of-life: Quality-of-life was defined as the change in quality-of-life scores from baseline to the available time points.

Patients' satisfaction: Patients' satisfaction was defined as the overall patient satisfaction scores or proportion of patients satisfied with their care at the available time points.

Caregivers' stress: Caregivers' stress was defined as the overall score on scale of caregiver.

Morbidity due to hospitalization: Morbidity due to hospitalization was defined as the number of adverse events, which included urinary tract infections (including catheter associated), falls, and standard hospital-acquired conditions at the available time points.

© 2021 Arsenault-Lapierre G et al. *JAMA Network Open.*

Cognitive status: Cognitive status was defined as the change in a cognitive status score at the available time points.

Nutrition status: Nutrition status was defined as the change in a nutrition score at the available time points.

Functional status: Functional status was defined as the change in a functional status score at the available time points.

Neurological deficit: Neurological status was defined as the change in a neurological status score at the available time points.

Efforts to obtain more information and data

To clarify methods, interventions and outcome definitions from studies and include as many studies as possible (and reduce heterogeneity), as well as to clarify or obtain more data, we contacted authors by email or system-based communication, up to three times, following methods described in Godard-Sebillotte et al. (2018). If we did not obtain this information, the data was considered incomplete and not included in our analyses.

We contacted the authors of five studies for additional information or data. We received clarifications and unpublished data on length of treatment in the HaH group for one study (Echevarria et al. 2018).

© 2021 Arsenault-Lapierre G et al. *JAMA Network Open.*

eAppendix 2. Data Transformation

**Outcome**	**Study**	**Data origin and/or transformation done before meta-analysis**
Readmission	Mendoza et al. (2009)<br>Ricauda et al. (2008)<br>Echevarria et al. (2018)<br>Tibaldi et al. (2009)<br>Davies et al. (2000)<br>Hernandez et al. (2002)	Number of observations was considered at baseline in order to account for readmissions from the beginning of the study until follow-up.

© 2021 Arsenault-Lapierre G et al. *JAMA Network Open.*

eTable 1. Justification for Narrative Synthesis

Outcome	Justification
Anxiety and depression	Two studies report using Geriatric Depression Scale: one reports mean, other reports median
Caregivers’ stress	Two studies report on Relative Stress Scale: one reports the change in score and standard deviation, other reports final scores and standard deviation
Functional status	Two studies use the Activities of Daily Living: one uses median, the other uses mean
Quality-of-life	Two studies report on St. George’s Respiratory Questionnaire: one has no standard deviation or confidence interval
Morbidity due to hospitalization	Four studies reported different morbidities and adverse events that were not comparable across studies

© 2021 Arsenault-Lapierre G et al. *JAMA Network Open.*

eTable 2. Justification for Sensitivity Analyses for Mortality

Original analysis	RCT excluded	Justification for exclusion	Results of sensitivity analyses	Accompanying forest plot in supplemental file 3
Suppl. 5, Figure A RR 95%CI: 0.61, 1.15	Vianello et al. (2013)	Younger population	RR 95%CI: 0.60, 1.15	Figure A: Forest plot of sensitivity analysis for mortality excluding the study with younger patients
	Hernandez et al. (2003); Tibaldi et al. (2009); Levine et al. (2018); Mendoza et al. (2009); Ricauda et al. (2008); Ricauda et al. (2004)	Follow-up period not 3 months	RR 95%CI: 0.42, 2.21	Figure B: Forest plot of sensitivity analysis for mortality excluding studies with follow-up period other than 3-month
	Davies et al. (2000); Hernandez et al. (2003); Vianello et al. (2013); Echevarria et al. (2018); Levine et al. (2018); Mendoza et al. (2009)	Follow-up period was not 6 months	RR 95%CI: 0.59, 1.22	Figure C: Forest plot of sensitivity analysis for mortality excluding studies with follow-up period other than 6-month follow-up
	Tibaldi et al. (2009); Vianello et al. (2013); Levine et al. (2018); Mendoza et al. (2009); Ricauda et al. (2004)	Not COPD	RR 95%CI: 0.46, 1.36	Figure D: Forest plot of sensitivity analysis for mortality excluding studies with patients other than those with chronic obstructive pulmonary disorders
	Davies et al. (2000); Hernandez et al. (2003); Vianello et al. (2013); Echevarria et al. (2018);	Not CHF	RR 95%CI: 0.39, 1.98	Figure E: Forest plot of sensitivity analysis for mortality

© 2021 Arsenault-Lapierre G et al. *JAMA Network Open.*

	Levine et al. (2018); Ricauda et al. (2008); Ricauda et al. (2004)			excluding studies with patients other than chronic heart failure

Legend

COPD: Chronic obstructive pulmonary disorder; CHF: Chronic heart failure; SD: standard deviation; RR: Risk Ratio; MD: Mean difference; CI: Confidence interval. *significance level changed from statistically significant (original analysis) to not statistically significant (sensitivity analysis).

© 2021 Arsenault-Lapierre G et al. *JAMA Network Open.*

eFigure 1. Forest Plots for Mortality Sensitivity Analyses

Figure A: Forest plot of sensitivity analysis for mortality excluding the study with younger patients

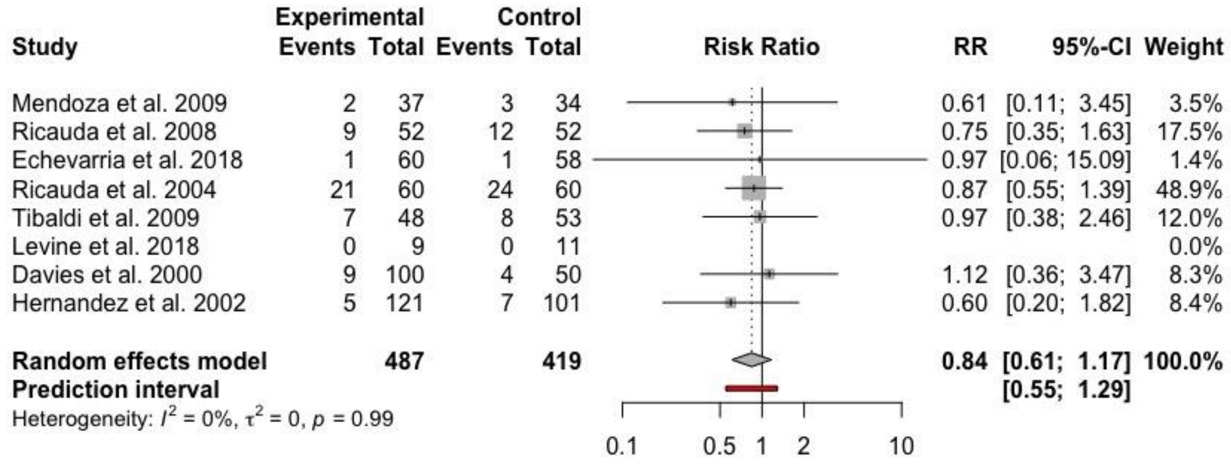

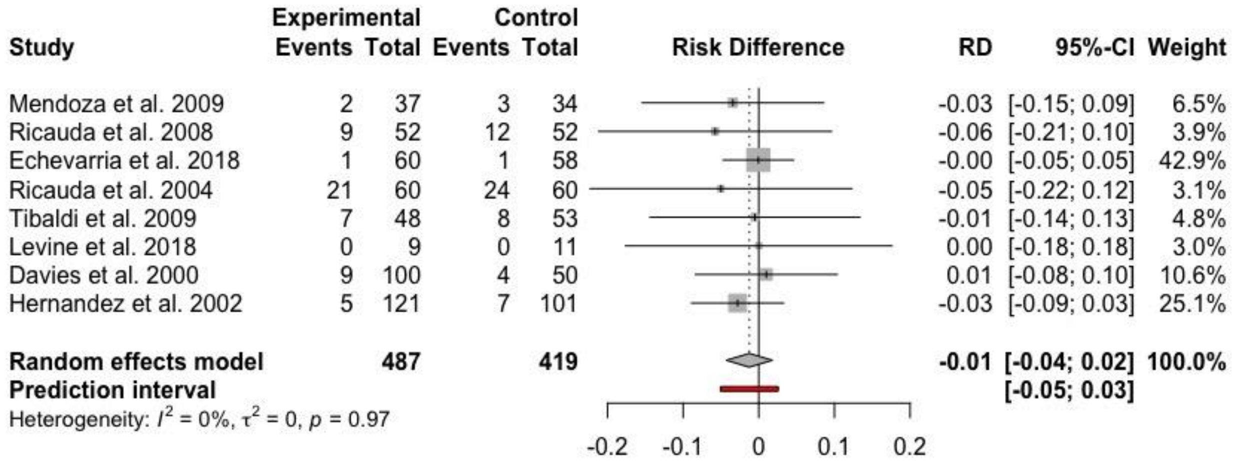

Legend
HaH: Hospital at Home; RR: Risk Ratio; RD: Risk Difference; CI: Confidence interval
τ^2: variance between studies; I^2: proportion of variance due to heterogeneity between studies.
Total number of observations used was sample size at baseline.

© 2021 Arsenault-Lapierre G et al. *JAMA Network Open.*

Figure B: Forest plot of sensitivity analysis for mortality excluding studies with follow-up period other than 3-month

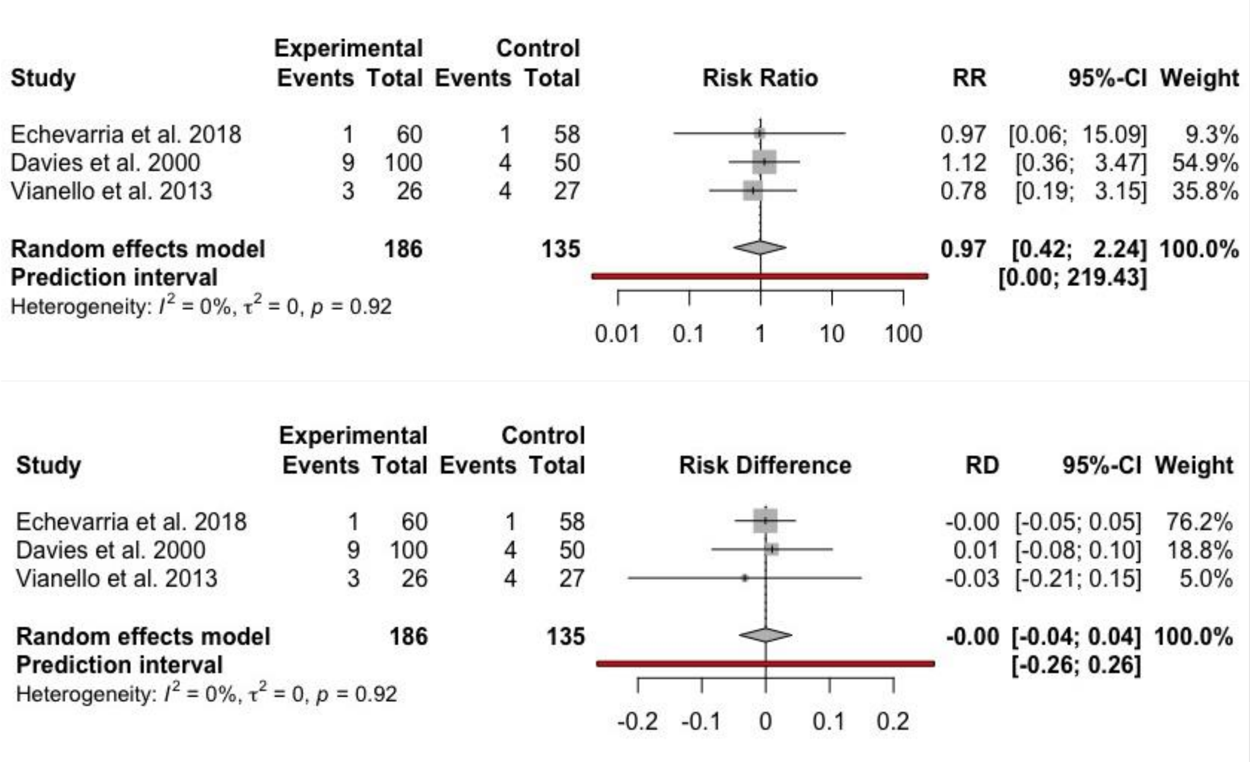

Legend
HaH: Hospital at Home; RR: Risk Ratio; RD: Risk Difference; CI: Confidence interval
τ^2: variance between studies; I^2: proportion of variance due to heterogeneity between studies.
Total number of observations used was sample size at baseline.

© 2021 Arsenault-Lapierre G et al. *JAMA Network Open.*

Figure C: Forest plot of sensitivity analysis for mortality excluding studies with follow-up period other than 6-month follow-up

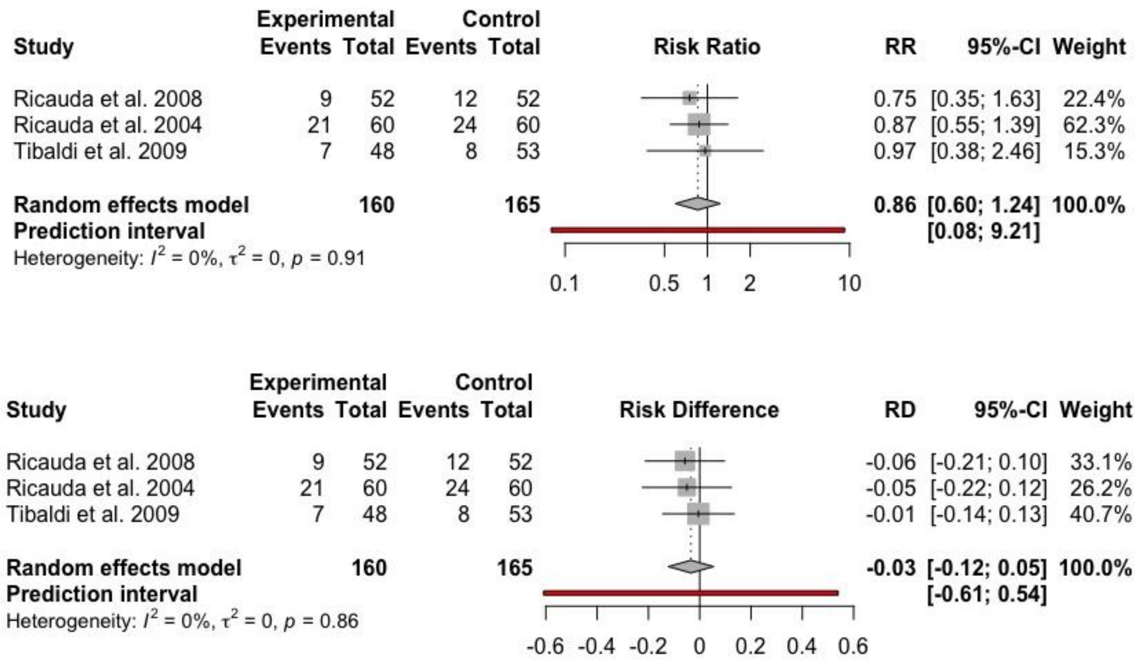

Legend

HaH: Hospital at Home; RR: Risk Ratio; RD: Risk Difference; CI: Confidence interval

τ^2: variance between studies; I^2: proportion of variance due to heterogeneity between studies.

Total number of observations used was sample size at baseline.

© 2021 Arsenault-Lapierre G et al. *JAMA Network Open.*

Figure D: Forest plot of sensitivity analysis for mortality excluding studies with patients other than those with chronic obstructive pulmonary disorders

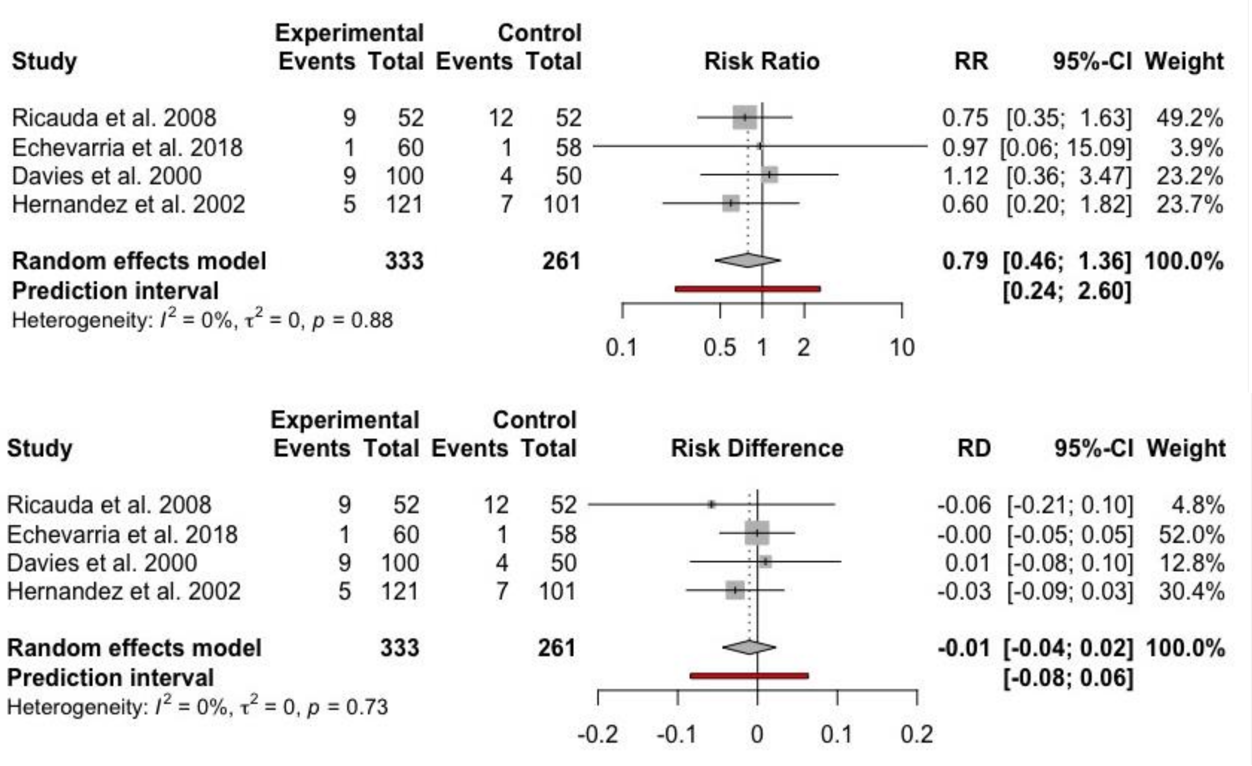

Legend
HaH: Hospital at Home; RR: Risk Ratio; RD: Risk Difference; CI: Confidence interval
τ^2: variance between studies; I^2: proportion of variance due to heterogeneity between studies.
Total number of observations used was sample size at baseline.

© 2021 Arsenault-Lapierre G et al. *JAMA Network Open.*

Figure E: Forest plot of sensitivity analysis for mortality excluding studies with patients other than chronic heart failure

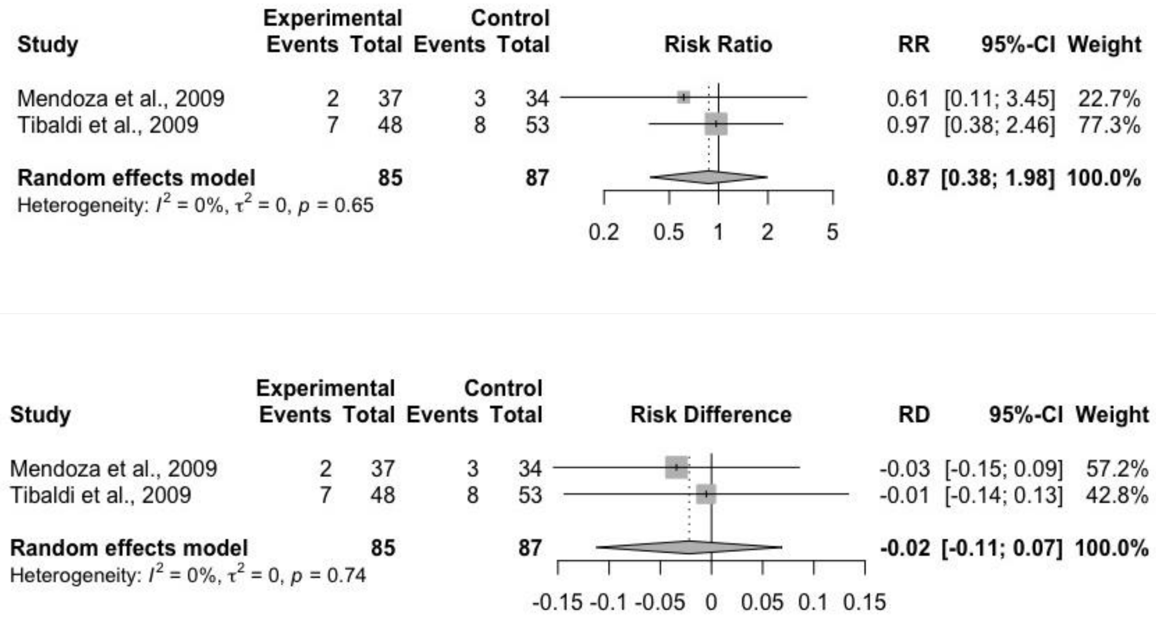

Legend
HaH: Hospital at Home; RR: Risk Ratio; RD: Risk Difference; CI: Confidence interval
τ^2: variance between studies; I^2: proportion of variance due to heterogeneity between studies.
Total number of observations used was sample size at baseline.

© 2021 Arsenault-Lapierre G et al. *JAMA Network Open.*

eTable 3. Justification of Sensitivity Analyses for Readmission

**Original analysis**	**RCT excluded**	**Justification for exclusion**	**Results of sensitivity analyses**	**Accompanying forest plot in supplemental file 3**
Suppl. 5, Figure B RR 95%CI: 0.55, 0.94	Tibaldi et al. (2009); Levine et al. (2018); Mendoza et al. (2009)	Not COPD	RR 95%CI: 0.55, 1.09*	eFigure 2. Forest Plots for Readmission Sensitivity Analyses Figure F: Forest plot of sensitivity analysis for readmission excluding studies with patients other than chronic obstructive pulmonary disorder
	Mendoza et al. (2009)	Considered only readmission due to CHF	RR 95%CI: 0.53, 0.98	Figure G: Forest plot of sensitivity analysis for readmission excluding studies with patients other than all-cause readmission
	Davies et al. (2000); Hernandez et al. (2003); Echevarria et al. (2018); Levine et al. (2018); Mendoza et al. (2009)	Follow-up period not 6 months	RR 95%CI: 0.34, 0.72	Figure H: Forest plot of sensitivity analysis for readmission excluding studies with a follow-up period other than 6 month
	Hernandez et al. (2003); Tibaldi et al. (2009); Levine et al. (2018); Mendoza et al. (2009); Ricauda et al. (2008)	Follow-up period not 3 months	RR 95%CI: 0.71, 1.37*	Figure I: Forest plot of sensitivity analysis for readmission excluding studies with a follow-up period other than 3 months

© 2021 Arsenault-Lapierre G et al. *JAMA Network Open.*

	Levine et al. (2018)	Small sample size and large variance	RR 95%CI: 0.58, 0.96	Figure J: Forest plot of sensitivity analysis for readmission excluding the study with a small sample sizeFigure K: Forest plot of sensitivity analysis for readmission excluding studies with patients other than those with chronic heart failure
	Davies et al. (2000); Hernandez et al. (2003); Echevarria et al. (2018); Levine et al. (2018); Ricauda et al. (2008)	Not CHF	RR 95%CI: 0.42, 1.10*	Figure K: Forest plot of sensitivity analysis for readmission excluding studies with patients other than those with chronic heart failure

Legend
RCT: Randomized Controlled Trial; COPD: Chronic Obstructive Pulmonary Disease; CHF: Chronic Heart Failure; RR: Risk Ratio; RD: Risk Difference; CI: Confidence interval.
*significance level changed from statistically significant (original analysis) to not statistically significant (sensitivity analysis).

© 2021 Arsenault-Lapierre G et al. *JAMA Network Open.*

eFigure 2. Forest Plots for Readmission Sensitivity Analyses

Figure F: Forest plot of sensitivity analysis for readmission excluding studies with patients other than chronic obstructive pulmonary disorder

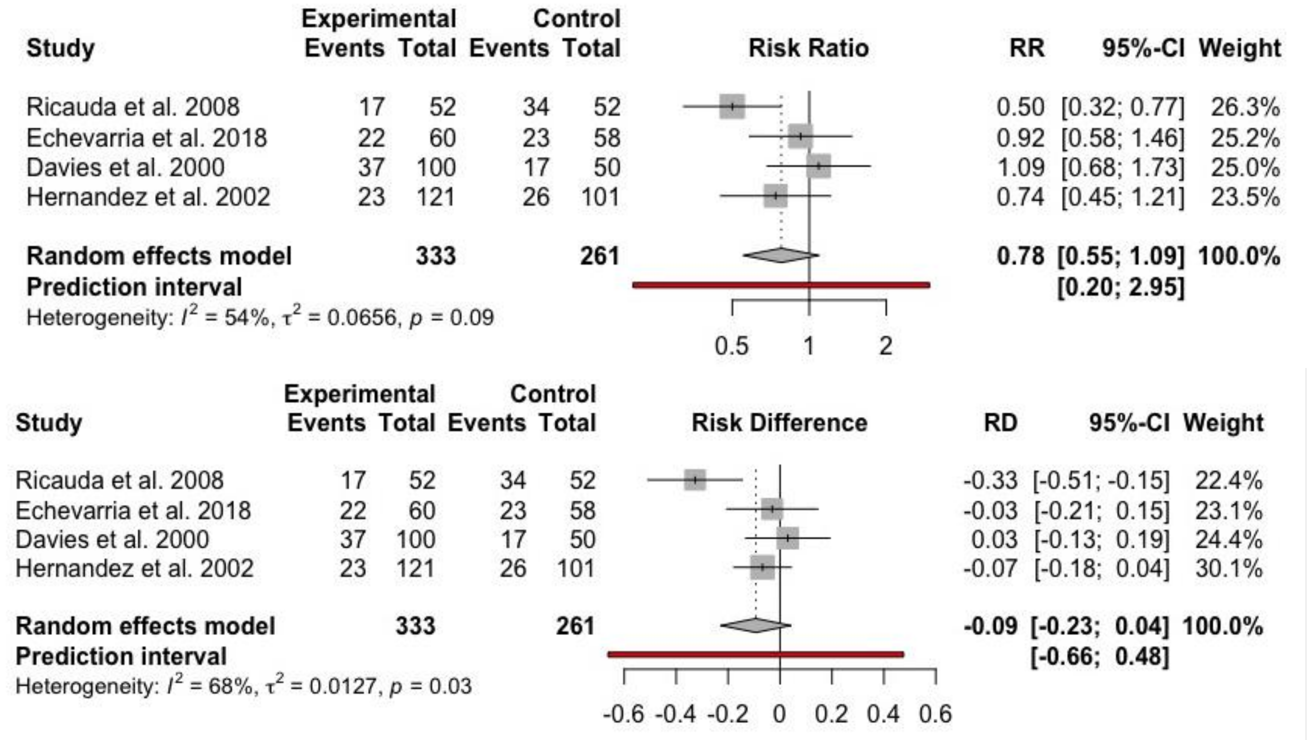

Legend
HaH: Hospital at Home; RR: Risk Ratio; RD: Risk Difference; CI: Confidence interval
τ^2: variance between studies; I^2: proportion of variance due to heterogeneity between studies.
Total number of observations used was sample size at baseline.

© 2021 Arsenault-Lapierre G et al. *JAMA Network Open.*

Figure G: Forest plot of sensitivity analysis for readmission excluding studies with patients other than all-cause readmission

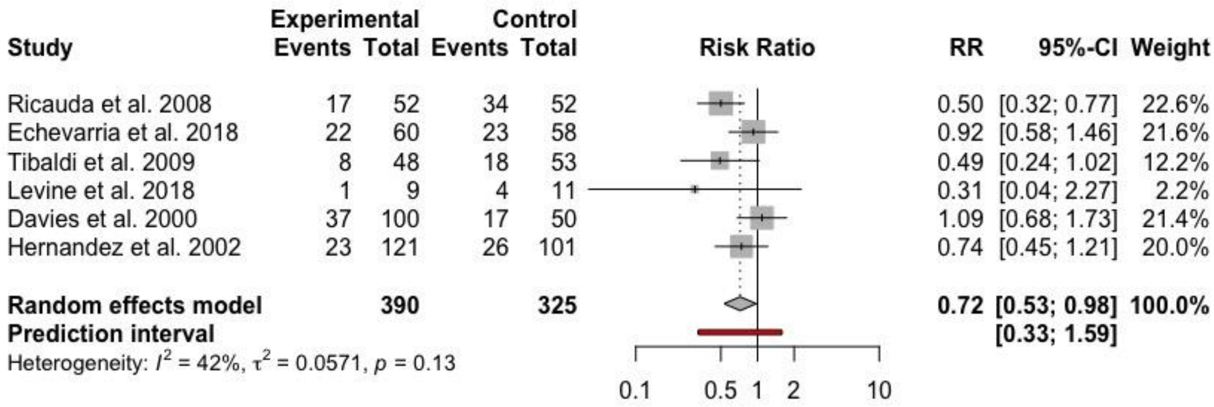

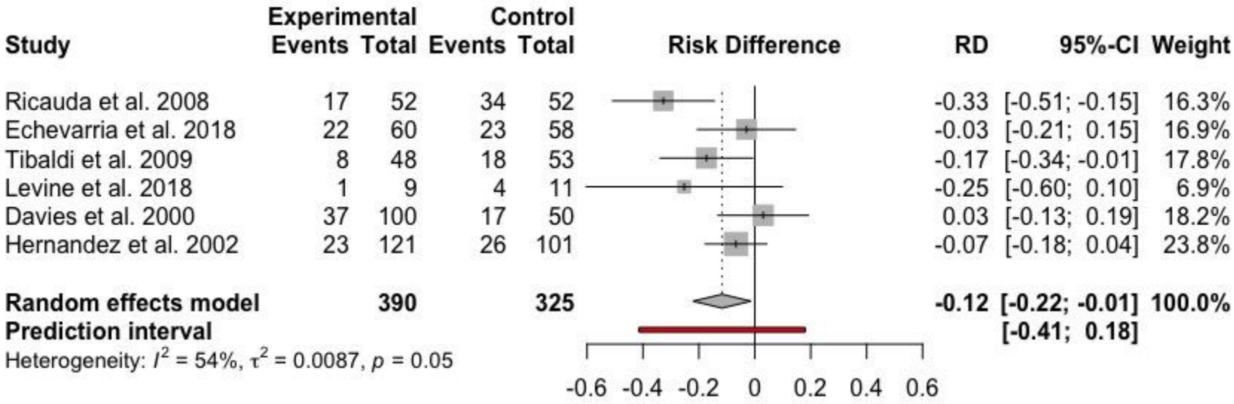

Legend
HaH: Hospital at Home; RR: Risk Ratio; RD: Risk Difference; CI: Confidence interval
τ^2: variance between studies; I^2: proportion of variance due to heterogeneity between studies.
Total number of observations used was sample size at baseline.

© 2021 Arsenault-Lapierre G et al. *JAMA Network Open.*

Figure H: Forest plot of sensitivity analysis for readmission excluding studies with a follow-up period other than 6 months

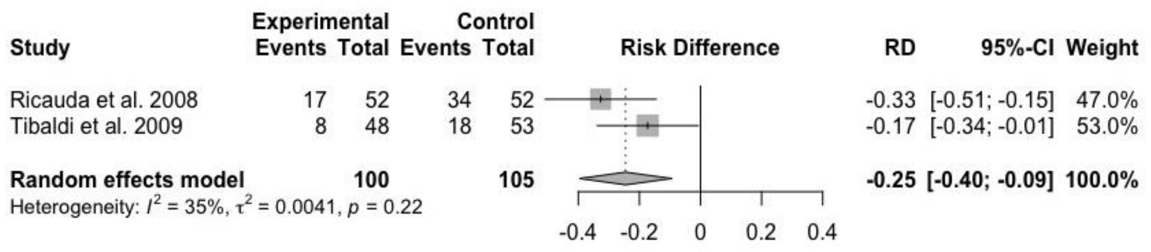

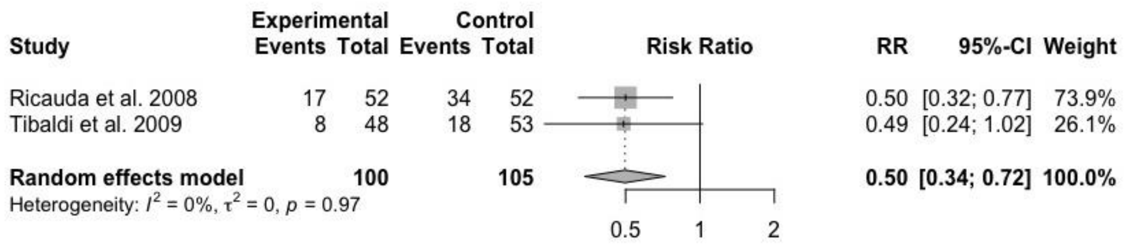

Legend
HaH: Hospital at Home; RR: Risk Ratio; RD: Risk Difference; CI: Confidence interval
τ^2: variance between studies; I^2: proportion of variance due to heterogeneity between studies.
Total number of observations used was sample size at baseline.

© 2021 Arsenault-Lapierre G et al. *JAMA Network Open.*

Figure I: Forest plot of sensitivity analysis for readmission excluding studies with a follow-up period other than 3 months

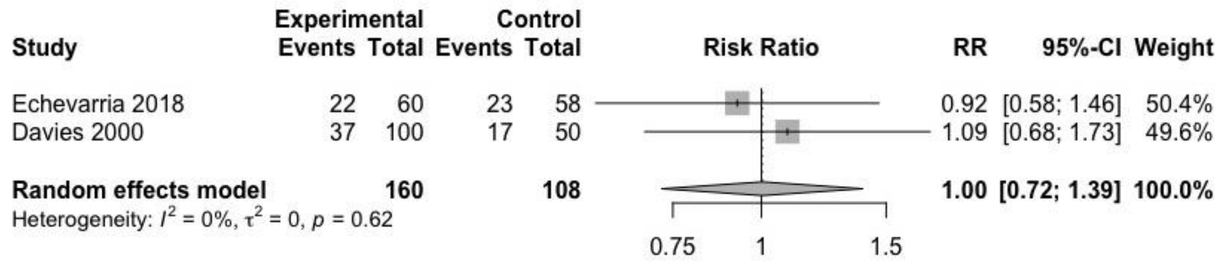

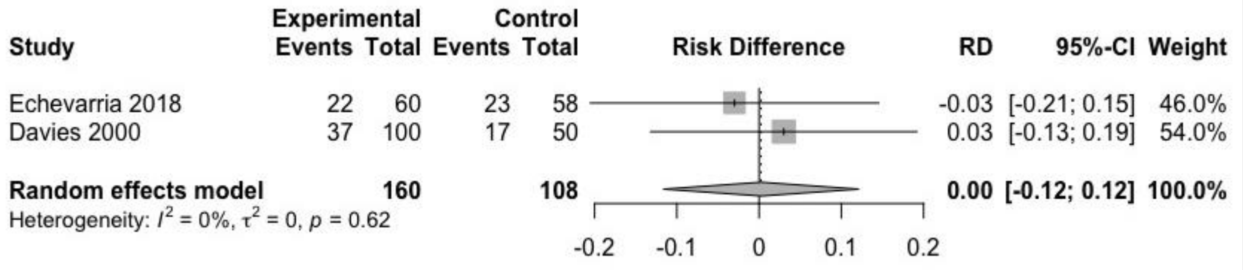

Legend
HaH: Hospital at Home; RR: Risk Ratio; RD: Risk Difference; CI: Confidence interval
τ^2: variance between studies; I^2: proportion of variance due to heterogeneity between studies.
Total number of observations used was sample size at baseline.

Figure J: Forest plot of sensitivity analysis for readmission excluding the study with a small sample size

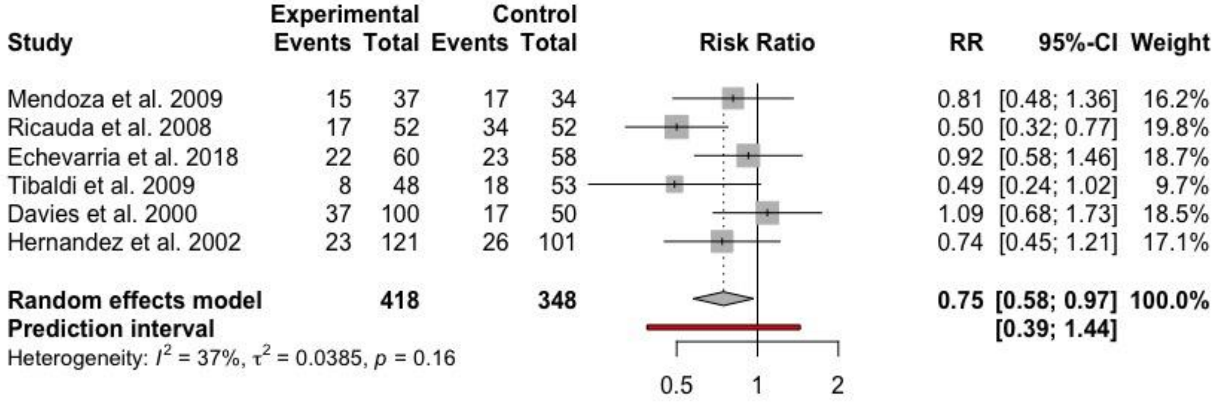

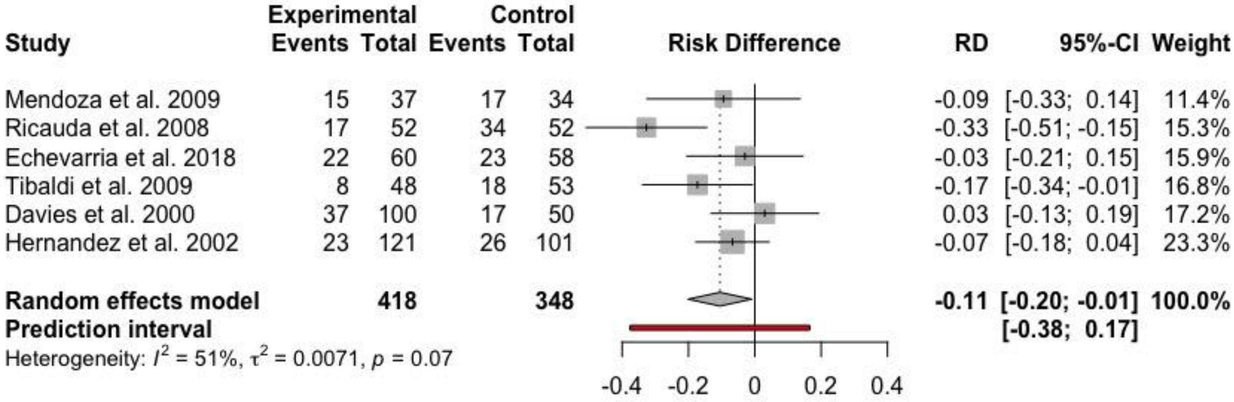

Legend
HaH: Hospital at Home; RR: Risk Ratio; RD: Risk Difference; CI: Confidence interval
τ^2: variance between studies; I^2: proportion of variance due to heterogeneity between studies.
Total number of observations used was sample size at baseline.

© 2021 Arsenault-Lapierre G et al. *JAMA Network Open.*

Figure K: Forest plot of sensitivity analysis for readmission excluding studies with patients other than those with chronic heart failure

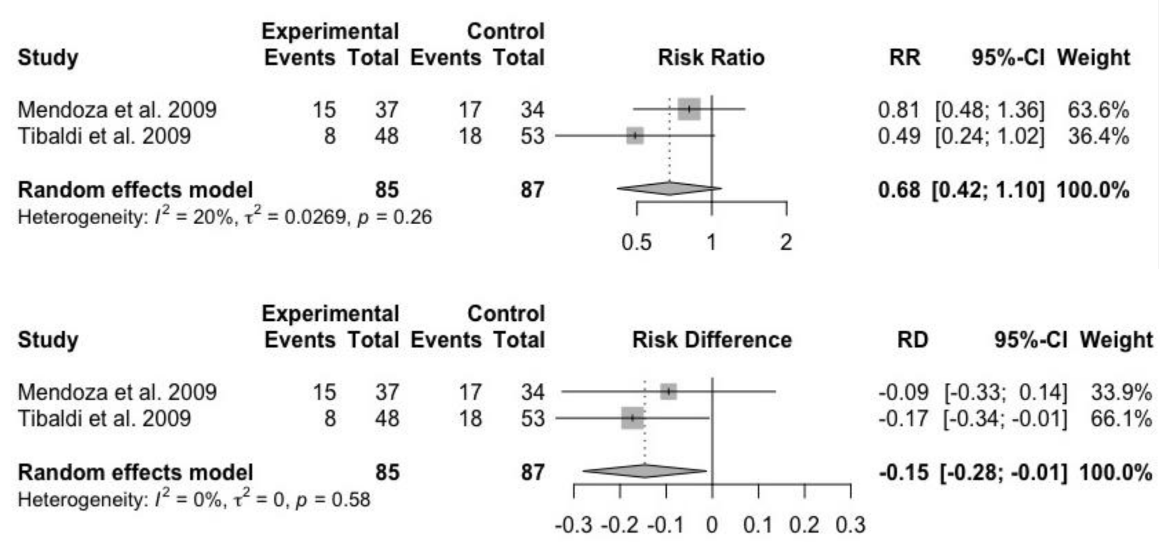

Legend
HaH: Hospital at Home; RR: Risk Ratio; RD: Risk Difference; CI: Confidence interval
τ^2: variance between studies; I^2: proportion of variance due to heterogeneity between studies.
Total number of observations used was sample size at baseline.

© 2021 Arsenault-Lapierre G et al. *JAMA Network Open.*

eTable 4. Justification of Sensitivity Analyses for Length of Treatment

**Original analysis**	**RCT excluded**	**Justification for exclusion**	**Results of sensitivity analyses**	**Accompanying forest plot in supplemental file 3**
Suppl. 5, Figure C RR 95%CI: 1.91, 8.98	Ricauda et al. (2008); Ricauda et al. (2004); Echevarria et al. (2018)	Not CHF	MD 95%CI: -0.08, 11.85*	Figure L: Forest plot of sensitivity analysis for length of treatment excluding studies with patients other than chronic heart failure
	Mendoza et al. (2009) Echevarria et al. (2018)	Follow-up period not 6 months	MD 95%CI: 3.63, 13.91	Figure M: Forest plot of sensitivity analysis for length of treatment excluding studies with follow-up period other than 6 months
	Ricauda et al. (2004); Tibaldi et al. (2009); Mendoza et al. (2009)	Not COPD	MD 95%CI: -1.18; 5.84*	Figure N: Forest plot of sensitivity analysis for length of treatment excluding studies with patients other than chronic obstructive pulmonary disorder

Legend
RCT: Randomized Controlled Trial; CHF: Chronic heart failure; MD: Mean difference; CI: Confidence interval; RR: Risk Ratio; COPD, Chronic obstructive pulmonary disorder.
*significance level changed from statistically significant (original analysis) to not statistically significant (sensitivity analysis).

© 2021 Arsenault-Lapierre G et al. *JAMA Network Open.*

eFigure 3. Forest Plots for Length of Treatment Sensitivity Analyses

Figure L: Forest plot of sensitivity analysis for length of treatment excluding studies with patients other than chronic heart failure

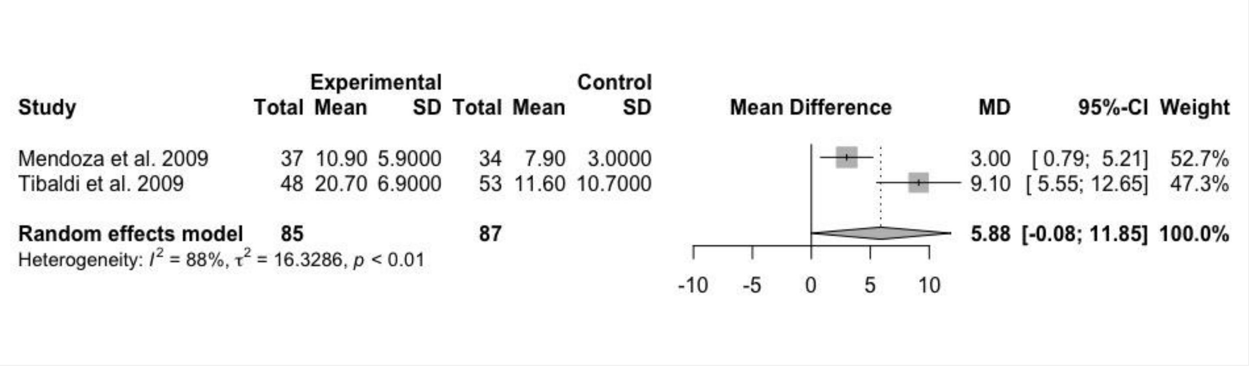

Legend

HaH: Hospital at Home; RR: Risk Ratio; RD: Risk Difference; CI: Confidence interval

τ^2: variance between studies; I^2: proportion of variance due to heterogeneity between studies.

Total number of observations used was sample size at baseline.

© 2021 Arsenault-Lapierre G et al. *JAMA Network Open.*

Figure M: Forest plot of sensitivity analysis for length of treatment excluding studies with follow-up period other than 6 months

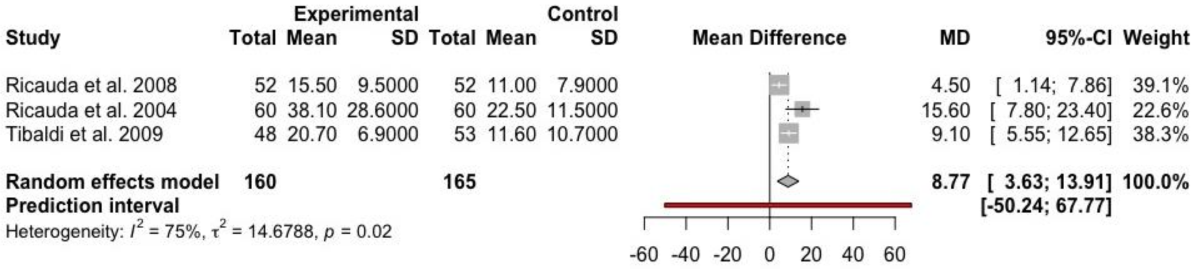

Legend
HaH: Hospital at Home; RR: Risk Ratio; RD: Risk Difference; CI: Confidence interval
τ^2: variance between studies; I^2: proportion of variance due to heterogeneity between studies.
Total number of observations used was sample size at baseline.

© 2021 Arsenault-Lapierre G et al. *JAMA Network Open.*

Figure N: Forest plot of sensitivity analysis for length of treatment excluding studies with patients other than chronic obstructive pulmonary disorder

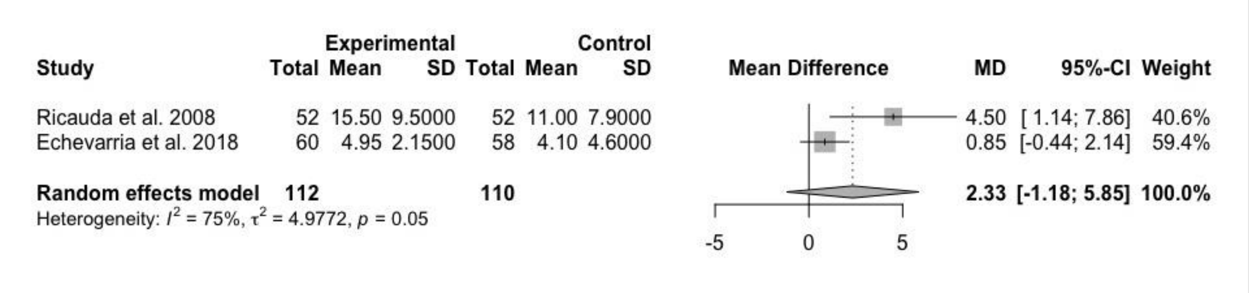

Legend

HaH: Hospital at Home; RR: Risk Ratio; RD: Risk Difference; CI: Confidence interval

τ^2: variance between studies; I^2: proportion of variance due to heterogeneity between studies.

Total number of observations used was sample size at baseline.

© 2021 Arsenault-Lapierre G et al. *JAMA Network Open.*

eAppendix 3. Summary of Findings Table

A) summary of meta-analyses findings

Author	Year	Country	Risk of bias (all studies had performance bias)	Chronic diseases	Nurse home visit	physician home visit	Length of follow-up (months)	HaH n	HaH mean age	HaH female %	Control n	Control mean age	Control female %	Mortality RR	Mortality 95%CI	Readmission RR	Readmission 95%CI	LoT MD	LoT 95%CI	LTC RR	LTC 95%CI
Hernandez et al.	2003	Spain	potential detection bias and potential reporting bias	COPD	yes	no	2	121	71	3	101	71	3	0,6	0.20-1.82	0,74	0.58-1.46				
Vianello et al.	2013	Italy	potential selection bias and potential detection bias	Neuromuscular disease	yes	no	3	26	45	35	27	47	11	0,78	0.19-3.15						
Echevarria et al.	2018	England	none other than performance bias	COPD	yes	no	3	60	71	53	58	69	52	0,97	0.06-15.09			0,85	-0.44-2.14		
Davies et al.	2000	England	detection bias and potential selection bias	COPD	yes	no	3	100	70	55	50	70	40	1,12	0.36-3.47	1,09	0.68-1.73				
Levine et al.	2018	United States	detection bias and potential reporting bias	CHF, COPD, or asthma	yes	yes	1	9	65*	22	11	60*	73			0,31	0.04-2.27				
Ricauda et al.	2008	Italy	none other than performance bias	COPD	yes	yes	6	52	80	44	52	79	25	0,75	0.35-1.63	0,5	0.32-0.77	4,5	1.14-7.86	0,06	0.00-1.33
Tibaldi et al.	2009	Italy	potential reporting bias	CHF	yes	yes	6	48	82	54	53	80	43	0,97	0.38-2.46	0,49	024-1.02	9,1	5.55-12.65	0,06	0.00-1.09
Mendoza et al.	2009	Spain	potential selection bias	CHF	yes	yes	12	37	78	54	34	80	29	0,61	0.11-3.45	0,81	0.48-1.36	3	0.79-5.21		
Ricauda et al.	2004	Italy	potential selection bias	Ischemic Stroke	yes	yes	6	60	83*	62	60	80*	48	0,87	0.55-1.39			15,6	7.80-23.40	0,5	0.05-5.37
**median**							**3,0**	**52,0**	**71,0**	**53,0**	**52,0**	**71,0**	**40,0**								
**pooled results**														**0.84**		**0.74**		**5.45**		**0.16**	
**pooled 95%CI**														**0.61-1.15**		**0.57-0.95**		**1.91-8.98**		**0.03-0.74**	
**pooled heterogeneity I2**														**0%**		**31%**		**87%**		**0%**	

B) summary of narrative synthesis findings

Author	Year	Country	Risk of bias (all studies had performance bias)	Chronic diseases	Nurse home visit	physician home visit	Length of follow-up (months)	HaH n	HaH mean age	HaH female %	Control n	Control mean age	Control female %	Cognitive status	Nutrition	Patient satisfaction	Morbidity due to hospitalization	Stress of caregiver	Anxiety and depression	Quality of life	Functional status	Neurological deficits
Hernandez et al.	2003	Spain	potential detection bias and potential reporting bias	COPD	yes	no	2	121	71	3	101	71	3			HaH > control				HaH > control		
Vianello et al.	2013	Italy	potential selection bias and potential detection bias	Neuromuscular disease	yes	no	3	26	45	35	27	47	11									
Echevarria et al.	2018	England	none other than performance bias	COPD	yes	no	3	60	71	53	58	69	52						HaH > control	HaH > control		
Davies et al.	2000	England	detection bias and potential selection bias	COPD	yes	no	3	100	70	55	50	70	40							No difference		
Levine et al.	2018	United States	detection bias and potential reporting bias	CHF, COPD, or asthma	yes	yes	1	9	65*	22	11	60*	73			No difference	HaH > control					
Ricauda et al.	2008	Italy	none other than performance bias	COPD	yes	yes	6	52	80	44	52	79	25	No difference	No difference	No difference	No difference	No difference	HaH > control	HaH > control	No difference	
Tibaldi et al.	2009	Italy	potential reporting bias	CHF	yes	yes	6	48	82	54	53	80	43				HaH > control	HaH > control				
Mendoza et al.	2009	Spain	potential selection bias	CHF	yes	yes	12	37	78	54	34	80	29							No difference	No difference	
Ricauda et al.	2004	Italy	potential selection bias	Ischemic Stroke	yes	yes	6	60	83*	62	60	80*	48				No difference		HaH > control		No difference	No difference
**median**							**3,0**	**52,0**	**71,0**	**53,0**	**52,0**	**71,0**	**40,0**									
**pooled results**																Mixed	Mixed	Mixed	HaH > control	Mixed	No difference	No difference

Legend: This table depicts an overview of each study's results, including patients and intervention characteristics, design and risk of bias appraisal, as well as the pooled average, median, relative risk (RR) or mean difference (MD), 95% confidence intervals (CI) and heterogeneity (I2) of the overall meta-analyses findings (part A) and the pooled results of the overall narrative synthesis findings (part B) of our systematic review. COPD stands for chronic obstructive. pulmonary disease, CHF stands for Chronic Heart Failure, HaH stands for Hosptial-at-home, LoT stands for lenght of treatment, and LTC stands for long-term care admission, finally 'HaH > control' signifies that the outcome under study is indicative of better health in HaH group than the in-hospital control group. The asterisks * denotes that median age was reported and is excluded from overall patient age median.

© 2021 Arsenault-Lapierre G et al. *JAMA Network Open.*

eAppendix 4. Forest Plots of Original Meta-Analyses

Figure A: Forest plots for mortality analyses in the HaH and in-hospital groups

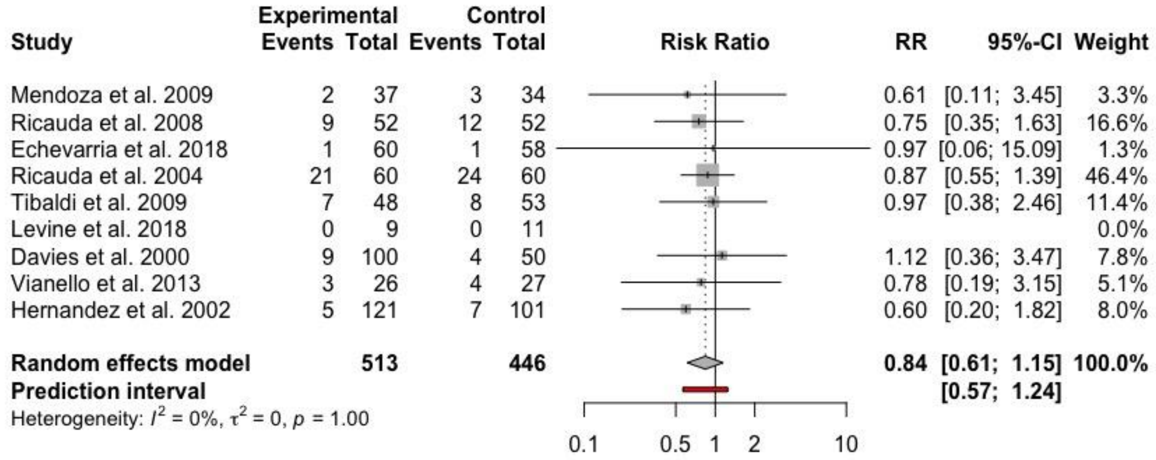

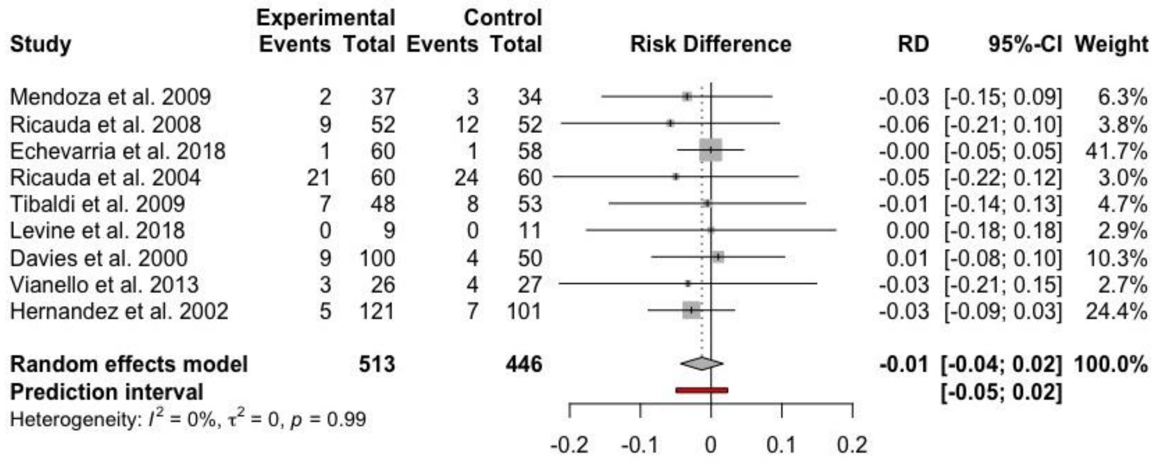

Legend

HaH: Hospital at Home; RR: Risk Ratio; RD: Risk Difference; CI: Confidence interval; τ^2: variance between studies; I^2: proportion of variance due to heterogeneity between studies. Total number of observations used was sample size at baseline.

© 2021 Arsenault-Lapierre G et al. *JAMA Network Open.*

Figure B: Forest plots for readmission in the HaH and in-hospital groups

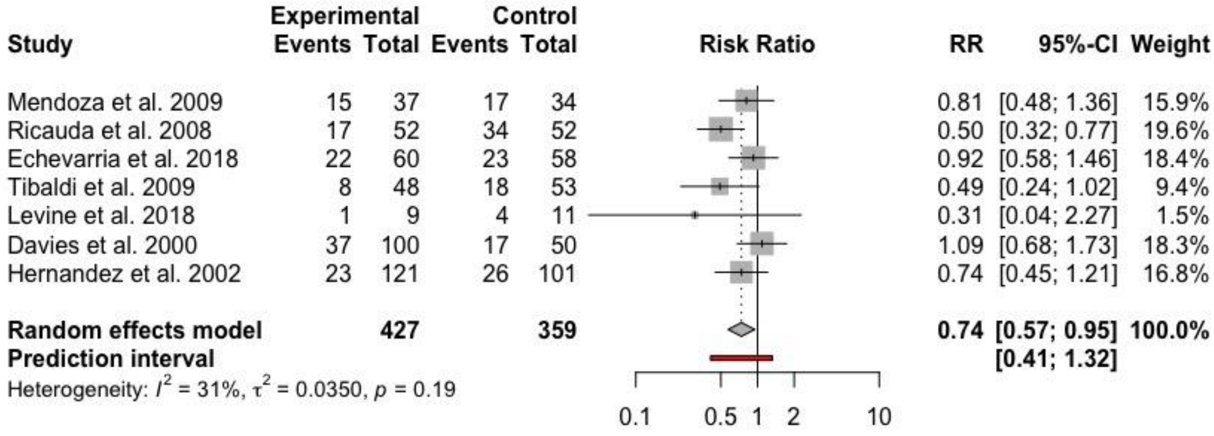

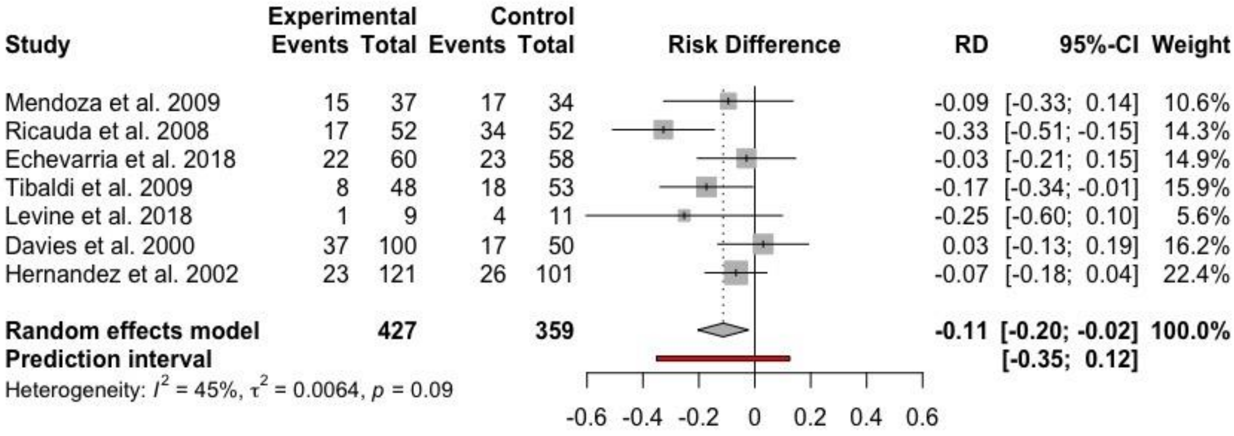

Legend

HaH: Hospital at Home; RR: Risk Ratio; RD: Risk Difference; CI: Confidence interval; τ^2: variance between studies; I^2: proportion of variance due to heterogeneity between studies. Total number of observations used was sample size at baseline.

© 2021 Arsenault-Lapierre G et al. *JAMA Network Open.*

Figure C: Forest plots for the length of treatment in the HaH and in-hospital groups

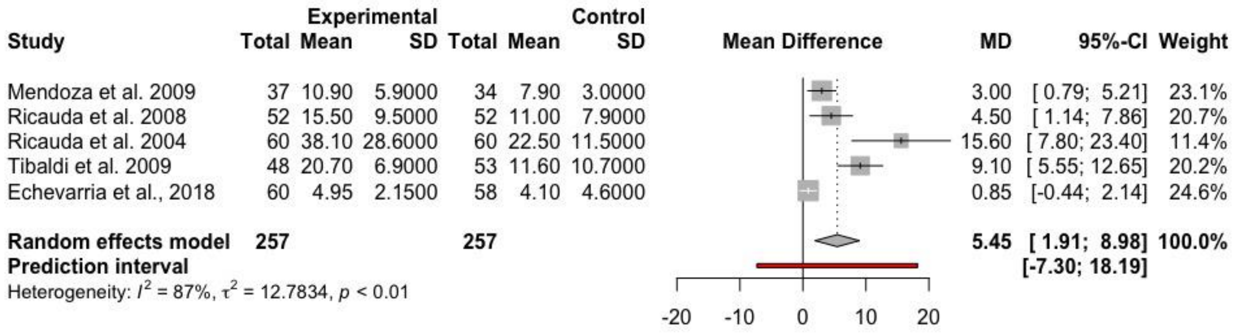

Legend
HaH: Hospital at Home; MD: Mean difference; CI: Confidence interval; SD: Standard deviation
τ^2: variance between studies; I^2: proportion of variance due to heterogeneity between studies.

© 2021 Arsenault-Lapierre G et al. *JAMA Network Open.*

Figure D: Forest plots for number of long-term facility admissions in the HaH and in-hospital groups

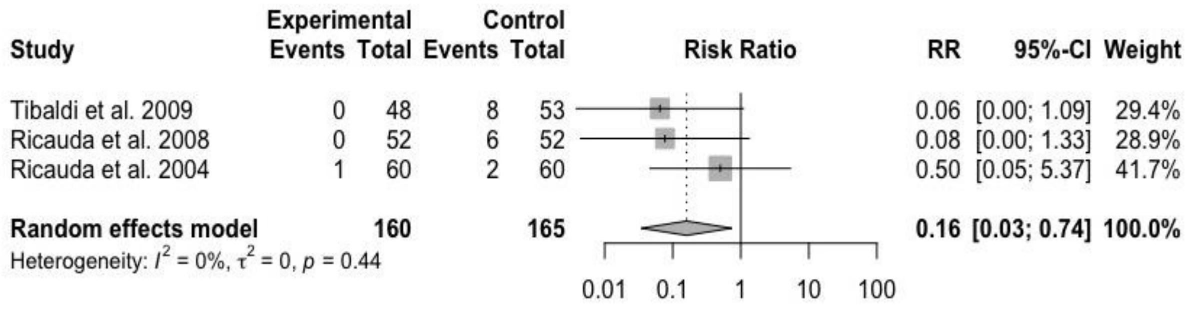

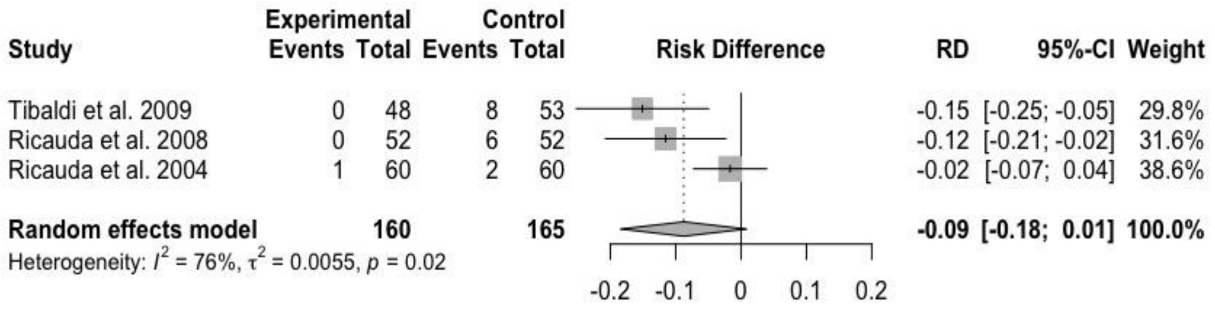

Legend
HaH: Hospital at Home; RR: Risk Ratio; RD: Risk Difference; CI: Confidence interval
τ^2: variance between studies; I^2: proportion of variance due to heterogeneity between studies.
Total number of observations used was sample size at baseline.

© 2021 Arsenault-Lapierre G et al. *JAMA Network Open.*

eReferences

Davies, L., M. Wilkinson, S. Bonner, P. M. Calverley, and R. M. Angus. 2000. '"Hospital at home" versus hospital care in patients with exacerbations of chronic obstructive pulmonary disease: prospective randomised controlled trial', *BMJ*, 321: 1265-8.

Echevarria, C., J. Gray, T. Hartley, J. Steer, J. Miller, A. J. Simpson, G. J. Gibson, and S. C. Bourke. 2018. 'Home treatment of COPD exacerbation selected by DECAF score: a non-inferiority, randomised controlled trial and economic evaluation', *Thorax*, 73: 713-22.

Godard-Sebillotte, Claire, Mélanie Le Berre, Sathya Karunananthan, Quan Nha Hong, and Isabelle Vedel. 2018. 'A digital media strategy to obtain unpublished data for a systematic review yields a very high author response rate', *J Clin Epidemiol*, 104: 141-43.

Hernandez, C., A. Casas, J. Escarrabill, J. Alonso, J. Puig-Junoy, E. Farrero, G. Vilagut, B. Collvinent, R. Rodriguez-Roisin, J. Roca, and Chronic project. 2003. 'Home hospitalisation of exacerbated chronic obstructive pulmonary disease patients', *Eur. Respir. J.*, 21: 58-67.

Levine, D. M., K. Ouchi, B. Blanchfield, K. Diamond, A. Licurse, C. T. Pu, and J. L. Schnipper. 2018. 'Hospital-Level Care at Home for Acutely Ill Adults: a Pilot Randomized Controlled Trial', *J Gen Intern Med*, 33: 729-36.

Mendoza, H., M. J. Martin, A. Garcia, F. Aros, F. Aizpuru, J. Regalado De Los Cobos, M. C. Bello, P. Lopetegui, and J. M. Cia. 2009. ''Hospital at home' care model as an effective alternative in the management of decompensated chronic heart failure', *EurJ Heart Fail*, 11: 1208-13.

Ricauda, N. A., M. Bo, M. Molaschi, M. Massaia, D. Salerno, D. Amati, V. Tibaldi, and F. Fabris. 2004. 'Home hospitalization service for acute uncomplicated first ischemic stroke in elderly patients: a randomized trial', *JAGS*, 52: 278-83.

Ricauda, NA., V. Tibaldi, B. Leff, C. Scarafiotti, R. Marinello, M. Zanocchi, and M. Molaschi. 2008. 'Substitutive "hospital at home" versus inpatient care for elderly patients with exacerbations of chronic obstructive pulmonary disease: a prospective randomized, controlled trial', *J Am Geriatr Soc*, 56: 493-500.

Tibaldi, V., G. Isaia, C. Scarafiotti, F. Gariglio, M. Zanocchi, M. Bo, S. Bergerone, and N. A. Ricauda. 2009. 'Hospital at home for elderly patients with acute decompensation of chronic heart failure: a prospective randomized controlled trial', *Arch Intern M*, 169: 1569-75.

Vianello, A., F. Savoia, E. Pipitone, B. Nordio, G. Gallina, L. Paladini, A. Concas, G. Arcaro, F. Gallan, and E. Pegoraro. 2013. '"Hospital at home" for neuromuscular disease patients with respiratory tract infection: a pilot study', *Respir Care*, 58: 2061-8.

© 2021 Arsenault-Lapierre G et al. *JAMA Network Open.*
